# Ancestry-Shift Refinement Mapping of the *C6orf97-ESR1* Breast Cancer Susceptibility Locus

**DOI:** 10.1371/journal.pgen.1001029

**Published:** 2010-07-22

**Authors:** Simon N. Stacey, Patrick Sulem, Carlo Zanon, Sigurjon A. Gudjonsson, Gudmar Thorleifsson, Agnar Helgason, Aslaug Jonasdottir, Soren Besenbacher, Jelena P. Kostic, James D. Fackenthal, Dezheng Huo, Clement Adebamowo, Temidayo Ogundiran, Janet E. Olson, Zachary S. Fredericksen, Xianshu Wang, Maxime P. Look, Anieta M. Sieuwerts, John W. M. Martens, Isabel Pajares, Maria D. Garcia-Prats, Jose M. Ramon-Cajal, Ana de Juan, Angeles Panadero, Eugenia Ortega, Katja K. H. Aben, Sita H. Vermeulen, Fatemeh Asadzadeh, K. C. Anton van Engelenburg, Sara Margolin, Chen-Yang Shen, Pei-Ei Wu, Asta Försti, Per Lenner, Roger Henriksson, Robert Johansson, Kerstin Enquist, Göran Hallmans, Thorvaldur Jonsson, Helgi Sigurdsson, Kristin Alexiusdottir, Julius Gudmundsson, Asgeir Sigurdsson, Michael L. Frigge, Larus Gudmundsson, Kristleifur Kristjansson, Bjarni V. Halldorsson, Unnur Styrkarsdottir, Jeffrey R. Gulcher, Kari Hemminki, Annika Lindblom, Lambertus A. Kiemeney, Jose I. Mayordomo, John A. Foekens, Fergus J. Couch, Olufunmilayo I. Olopade, Daniel F. Gudbjartsson, Unnur Thorsteinsdottir, Thorunn Rafnar, Oskar T. Johannsson, Kari Stefansson

**Affiliations:** 1 deCODE Genetics, Reykjavik, Iceland; 2 Department of Medicine and Center for Clinical Cancer Genetics, University of Chicago, Chicago, Illinois, United States of America; 3 Division of Oncology, Department of Surgery, College of Medicine, University of Ibadan, University College Hospital, Ibadan, Oyo, Nigeria; 4 Department of Laboratory Medicine and Pathology and Department of Health Sciences Research, Mayo Clinic, Rochester, Minnesota, United States of America; 5 Department of Medical Oncology, Erasmus MC Rotterdam, Josephine Nefkens Institute and Cancer Genomics Center, Rotterdam, The Netherlands; 6 Division of Medical Oncology, University Hospital, Zaragoza, Spain; 7 Divisions of Surgical Pathology and Gynecology, San Jorge Hospital, Huesca, Spain; 8 Division of Medical Oncology, Marques de Valdecilla University Hospital, Santander, Spain; 9 Division of Medical Oncology, Hospital Ciudad de Coria, Coria, Spain; 10 Division of Medical Oncology, University Hospital, Lérida, Spain; 11 Comprehensive Cancer Centre IKO, Nijmegen, The Netherlands; 12 Department of Epidemiology, Biostatistics and Health Technology Assessment, Radboud University Nijmegen Medical Centre, Nijmegen, The Netherlands; 13 Department of Human Genetics, Radboud University Nijmegen Medical Centre, Nijmegen, The Netherlands; 14 Department of Surgery, Slingeland Hospital, Doetinchem, The Netherlands; 15 Department of Oncology and Pathology, Karolinska Institute, Stockholm, Sweden; 16 Institute of Biomedical Sciences, Academia Sinica, Taipei, Taiwan; 17 Graduate Institute of Environmental Science, China Medical University, Taichung, Taiwan; 18 Division of Molecular Genetic Epidemiology, German Cancer Research Center, Heidelberg, Germany; 19 Center for Primary Health Care Research, Clinical Research Center, Lund University, Malmö, Sweden; 20 Department of Oncology, Norrlands University Hospital, Umeå, Sweden; 21 Department of Public Health and Clinical Medicine/Nutritional Research, Umeå University, Umeå, Sweden; 22 Departments of Oncology, Surgery, and The Cancer Center, Landspitali-University Hospital, Reykjavik, Iceland; 23 Department of Molecular Medicine and Surgery, Karolinska Institute, Stockholm, Sweden; 24 Department of Urology, Radboud University Nijmegen Medical Centre, Nijmegen, The Netherlands; 25 Health Science Institute, Nanotechnology Institute of Aragon, Zaragoza, Spain; University of Oxford, United Kingdom

## Abstract

We used an approach that we term ancestry-shift refinement mapping to investigate an association, originally discovered in a GWAS of a Chinese population, between rs2046210[T] and breast cancer susceptibility. The locus is on 6q25.1 in proximity to the *C6orf97* and estrogen receptor α (*ESR1*) genes. We identified a panel of SNPs that are correlated with rs2046210 in Chinese, but not necessarily so in other ancestral populations, and genotyped them in breast cancer case∶control samples of Asian, European, and African origin, a total of 10,176 cases and 13,286 controls. We found that rs2046210[T] does not confer substantial risk of breast cancer in Europeans and Africans (OR = 1.04, *P* = 0.099, and OR = 0.98, *P* = 0.77, respectively). Rather, in those ancestries, an association signal arises from a group of less common SNPs typified by rs9397435. The rs9397435[G] allele was found to confer risk of breast cancer in European (OR = 1.15, *P* = 1.2×10^−3^), African (OR = 1.35, *P* = 0.014), and Asian (OR = 1.23, *P* = 2.9×10^−4^) population samples. Combined over all ancestries, the OR was 1.19 (*P* = 3.9×10^−7^), was without significant heterogeneity between ancestries (*P_het_* = 0.36) and the SNP fully accounted for the association signal in each ancestry. Haplotypes bearing rs9397435[G] are well tagged by rs2046210[T] only in Asians. The rs9397435[G] allele showed associations with both estrogen receptor positive and estrogen receptor negative breast cancer. Using early-draft data from the 1,000 Genomes project, we found that the risk allele of a novel SNP (rs77275268), which is closely correlated with rs9397435, disrupts a partially methylated CpG sequence within a known CTCF binding site. These studies demonstrate that shifting the analysis among ancestral populations can provide valuable resolution in association mapping.

## Author Summary

In genome-wide association studies of disease susceptibility, there is no particular expectation that a genotyped SNP showing an association is itself a pathogenic variant. Rather, it is more likely that a SNP giving a signal does so because it is in linkage disequilibrium (LD) with a pathogenic variant. When the analysis is shifted to a population of another ancestry, the tagging relationship between the genotyped SNP and the pathogenic variant may be disrupted, due to differing patterns of LD between populations. Thus, it is not straightforward to determine whether a susceptibility locus identified in one ancestral population is also associated with risk in another. Moreover, the differing patterns of LD between ancestral populations can be used to gain resolution in genetic mapping. We refer to this approach as ancestry-shift refinement mapping. Here, we apply it to a breast cancer risk variant near the estrogen receptor α gene that was initially described in a Chinese population. We show that the tagging relationship between the originally described SNP rs2046210 and the pathogenic variant(s) is not maintained in Europeans and Africans. We identify a SNP, rs9397435, that is associated with breast cancer risk in populations of Asian, European, and African ancestry.

## Introduction

Recent genome-wide association studies (GWAS) have identified a number of new susceptibility loci for breast cancer and other cancers [1]–[5]. In most studies, strong evidence has been obtained for risk association in one particular ancestral group, usually Europeans. SNPs represented on microarray chips used in GWAS protocols are selected in part because they each tag a group of correlated, ungenotyped SNPs through linkage disequilibrium (LD). There is no particular expectation that a SNP identified in a GWAS is a pathogenic, causative variant. Rather, it is more likely that such a SNP is in LD with a pathogenic variant (or a set of pathogenic variants, see *Discussion*) that is not genotyped directly. If the analysis is moved to a population with different ancestry, then the tagging relationship between the SNP and the pathogenic variant may be disrupted as a result of the difference in LD between ancestral populations [6].

There are two main motivations for identifying SNPs whose property of tagging a pathogenic variant is not disrupted by changes in LD resulting from a shift to another ancestral population. Firstly, one might wish to test for risk arising from the susceptibility locus in a population of different ancestry, or indeed to determine whether a similar pathogenic variant exists at the susceptibility locus in another ancestral group. Secondly, by moving the analysis into another ancestral population, it might be possible to separate SNPs that are so highly correlated in the original population that their risk associations are indistinguishable. This could aid in the identification of SNPs that are most strongly correlated with the pathogenic variant, and move the analysis closer to the identification of the pathogenic variant itself. This approach, which we term ancestry-shift refinement mapping, has been formalized and used previously [5], [7]–[12]. However the interpretation has sometimes been limited by low power in the target ancestral populations or the lack of comprehensive genotypes.

**Figure 1:**
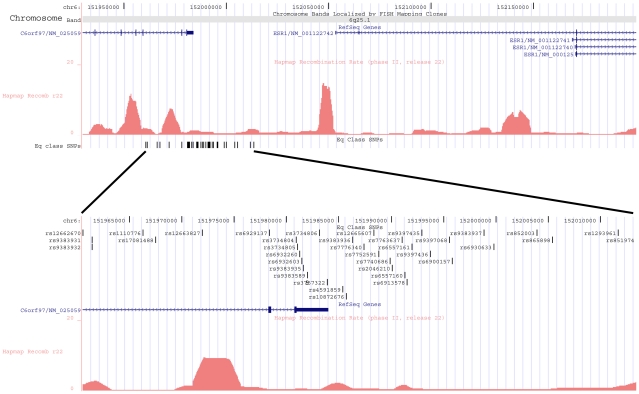
Overview of the *C6orf97-ESR1* breast cancer susceptibility locus. The upper panel shows a view of the genomic region of chromosome 6, nucleotides 151,930,000–152,200,000 taken from the UCSC browser Build 36 assembly (hg18). The *C6orf97* gene and the four RefSeq isoforms of *ESR1* are shown. Below them is a histogram of the local recombination rates calculated as previously described [38] from HapMap phase II release 22 data. Below that is a track showing the locations of the SNPs that are correlated (r^2^≥0.65) with rs2046210 in Han Chinese (“Eq Class SNPs”). The lower panel shows a zoomed view of the region nucleotides 151,960,548–152,013,381, containing the Eq Class SNPs. RefSeq genes and recombination rates are as in the upper panel.

The estrogen receptor α (*ESR1*) locus has been a focus of attention because of the roles of estrogen in risk of breast cancer, osteoporosis and other conditions. Moreover, estrogen receptor (ER) expression in breast tumours is of prime prognostic importance [13]. Many investigations have been conducted searching for risk associations with sequence variants in *ESR1*, generally with equivocal results [14]. Comprehensive tag-SNP and meta-analyses found little evidence of breast cancer risk variants in the *ESR1* gene itself [15], [16]. Recently, a GWAS conducted in a large sample from the Shanghai Breast Cancer Study identified an association between rs2046210 and breast cancer in Chinese [1]. SNP rs2046210 is located 180kb 5′ to the major *ESR1* transcript initiation sites (and 63kb 5′ to the start site of *ESR1* isoform 4). The SNP is about 6kb downstream of the 3′ end of *C6orf97*, a RefSeq gene of unknown function (Figure 1, upper panel). The LD structure of the region is shown in Figure S1. Zheng et al. [1] reported that rs2046210 also confers risk of breast cancer in a population of European ancestry (allelic OR = 1.15, *P* = 0.01). However, the evidence from the publically available CGEMS dataset is more equivocal [17]. We estimated from the CGEMS data an allelic OR of 1.09, *P* = 0.25 for rs6900157, the best tagger (r^2^ = 0.93 in HapMap CEU) of rs2046210 on the CGEMS Illumina chip. Our own breast cancer GWAS dataset from 1,982 patients and 35,895 controls [4], [5] provided no evidence of a risk associated with rs6900157 in Europeans (allelic OR = 1.04, *P* = 0.36).

We suspected that the reason for our failure to replicate the Zheng et al. signal in Europeans could be because the LD relationship between the reported SNP rs2046210 and the pathogenic variant(s) might differ between Chinese and Europeans. Here we show that this is indeed the case. By studying a large class of SNPs that are highly correlated in Chinese but not necessarily so in ancestral Europeans and Africans, we were able to identify a class of less common SNPs (6–7% minor allele frequency [MAF] in Europeans and 1–6% in Africans) that are associated with breast cancer risk in non-Asian populations. The most strongly associated SNP, rs9397435, fully accounts for the association in all three ancestries.

## Results

**Figure 2:**
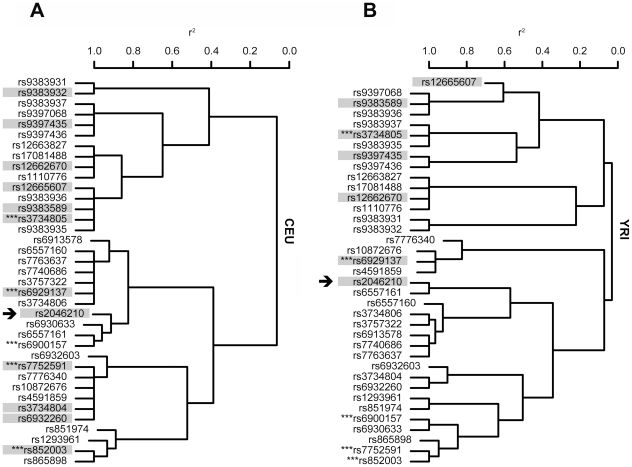
Dendrograms showing r^2^ relationships between *C6orf97-ESR1* SNPs in Europeans (CEU) and Yoruba Africans (YRI). On the left are listed the 37 SNPs that are correlated with an r^2^≥0.65 with rs2046210 (arrowed) in HapMap Han Chinese (CHB). In panel (a) the SNPs are arranged in a hierarchical cluster dendrogram based on the r^2^ values between them in the HapMap CEU sample of a European ancestry population. SNPs that were selected for genotyping are highlighted. SNPs indicated by *** are present on the Illumina Human Hap300 or HumanCNV370 chips used in the Icelandic GWAS. Panel (b) shows the same SNPs in a dendrogram based on r^2^ values from Yoruba Africans (HapMap YRI). Data are derived from HapMap Phase II release 23a.

To search for SNPs that might detect the *C6orf97-ESR1* signal in non-Asian ancestries, we first identified 36 SNPs that are well correlated (r^2^≥0.65) with rs2046210 in the Chinese, using the HapMap CHB dataset (Figure 1, lower panel, Figure S2). Then, using the HapMap CEU dataset, we observed the pattern of correlations between these SNPs in a population of European ancestry. The dendrogram in Figure 2A shows a hierarchical clustering of the 37 SNPs, based on their r^2^ values. We defined equivalence classes as sets of SNPs (or branches of the dendrogram) that show a correlation with an r^2^≥0.8 in CEU. We then selected a set of SNPs for genotyping such that at least one SNP in each equivalence class was included. These SNPs are highlighted in Figure 2A. We put in some redundant SNPs, partly to cover additional class fractionation in Africans (see below), and partly in order to examine two non-synonymous coding SNPs in the *C6orof97* gene: V604I (rs6929137) and V683I (rs3734804). Single track Centaurus [18] assays were generated for the selected SNPs and validated by typing them in the HapMap CEU, CHB/JPT, and YRI samples.

We then typed the selected SNPs in a series of breast cancer case∶control samples of European ancestry from Iceland, the Netherlands, Spain, Sweden, and U.S.A.; a total of 7,899 breast cancer cases and 11,234 controls. Details of the sample sets are given in Table S1. In addition, we typed the selected SNPs in a sample of 1,126 breast cancer cases and 1,118 controls of Chinese ancestry from Taiwan. The results are summarized in Table 1 and individual results for each sample set are given in Table S2. We used a likelihood approach to ensure that the same individuals were tested for each SNP, so that the *P* values for the different SNPs could be compared directly (see *Materials and Methods*). The results from the Taiwanese sample confirmed the association between breast cancer risk and the key SNP rs2046210 (OR = 1.24, *P* = 4.3×10^−4^) that was previously reported in Shanghai Chinese [1]. We also obtained significant signals for a range of SNPs that are highly correlated with rs2046210 in the Taiwanese. However, in the combined European ancestry populations, it was evident that rs2046210 confers little or no risk of breast cancer (OR = 1.04, *P* = 0.099, Table 1), despite the fact that the MAF of rs2046210 is almost the same in Asians and Europeans. However, we observed significant risk estimates in Europeans arising from a group of SNPs with MAFs in the 6–7% range, tagged by rs9397435, rs12662670, rs12665607, rs9383589 and rs3734805 (Table 1). The association *P* values for these SNPs remained significant if we applied Bonferroni correction for the number of European equivalence classes tested (significance threshold *P* = 0.05 divided by 7 classes = 0.007). These SNPs are highly or moderately correlated with each other in Europeans, judging from the HapMap data (Figure 2A) and the observed data from the genotyped samples (Figure S3). The strongest signal came from rs9383589[G] (OR = 1.15, *P* = 6.2×10^−4^). Thus, if the pathogenic variant that is present in Chinese is also present in Europeans, then in Europeans it appears to be tagged better by rs9383589 than by rs2046210. There was no substantial signal detected from either of the two coding variants in *C6orf97* (rs6929137 and rs3734804), ruling them out as causative variants (Table 1). It should be noted that this analysis does not represent a comprehensive scan for risk variants at the locus in Europeans, but is limited to SNPs that are strongly associated with the signal in Asians.

**Table 1:** Association of C6orf97/ESR1 SNPs with breast cancer in populations of different ancestries.

SNP	Allele	Ancestry^a^	Frequency in Controls^b^	OR	95% CI	*P*
rs9383932	G	Asian	0.378	1.25	(1.11, 1.41)	2.9×10^−4^
		European	0.132	1.06	(1.00, 1.13)	0.066
rs9397435	G	Asian	0.326	1.23	(1.09, 1.40)	8.0×10^−4^
		European	0.063	1.15	(1.06, 1.25)	1.2×10^−3^
		African & African American	0.063	1.35	(1.06, 1.71)	0.014
		All Ancestries	NA	1.19	(1.11, 1.27)	3.9×10^−7^
rs12662670	G	Asian	0.343	1.22	(1.07, 1.39)	2.5×10^−3^
		European	0.071	1.12	(1.03, 1.21)	6.4×10^−3^
		African & African American	0.027	1.54	(1.06, 2.23)	0.022
		All Ancestries	NA	1.16	(1.08, 1.24)	1.9×10^−5^
rs12665607	A	Asian	0.323	1.24	(1.10, 1.40)	6.2×10^−4^
		European	0.072	1.14	(1.05, 1.23)	1.1×10^−3^
		African & African American	0.010	1.94	(1.14, 3.30)	0.015
		All Ancestries	NA	1.18	(1.10, 1.26)	1.2×10^−6^
rs9383589	G	Asian	0.323	1.20	(1.06, 1.36)	4.2×10^−3^
		European	0.070	1.15	(1.06, 1.25)	6.2×10^−4^
		African & African American	0.016	1.61	(1.05, 2.48)	0.029
		All Ancestries	NA	1.17	(1.10, 1.25)	2.5×10^−6^
rs3734805	C	Asian	0.324	1.22	(1.07, 1.37)	2.0×10^−3^
		European	0.072	1.13	(1.05, 1.23)	1.8×10^−3^
		African & African American	0.028	1.27	(0.91, 1.78)	0.16
		All Ancestries	NA	1.16	(1.09, 1.23)	7.8×10^−6^
rs6929137	A	Asian	0.348	1.15	(1.02, 1.30)	0.025
		European	0.316	1.04	(0.99, 1.09)	0.082
		African & African American	0.538	1.02	(0.89, 1.16)	0.81
		All Ancestries	NA	1.05	(1.01, 1.09)	0.017
rs2046210	T^c^	Asian	0.363	1.24	(1.10, 1.40)	4.3×10^−4^
		European	0.337	1.04	(0.99, 1.08)	0.099
		African & African American	0.716	0.98	(0.86, 1.11)	0.77
		All Ancestries	NA	1.06^d^	(1.01, 1.10)	9.8×10^−3^
rs7752591	A	Asian	0.421	1.25	(1.11, 1.40)	3.0×10^−4^
		European	0.495	1.03	(0.99, 1.07)	0.16
rs3734804	A	Asian	0.422	1.21	(1.08, 1.37)	1.4×10^−3^
		European	0.474	1.00	(0.95, 1.05)	0.97
rs6932260	C	Asian	0.422	1.23	(1.09, 1.38)	7.7×10^−4^
		European	0.474	1.00	(0.95, 1.05)	0.96
rs852003	A	Asian	0.424	1.26	(1.12, 1.42)	1.2×10^−4^
		European	0.556	1.04	(0.99, 1.08)	0.11

^**a**^Numbers of Cases and Controls are Asian: 1,126 cases and 1,118 controls; European: 7,899 cases and 11,234 controls; African & African American: 1,151 cases and 934 controls; All Ancestries: 10,176 cases and 13,286 controls. Details of the sample sets for each ancestry are in Table S1 and association data for each individual sample set are in Table S2.

^**b**^Quoted Frequency in controls is the simple arithmetic average of all European sample sets for European Ancestry and the frequency in Nigerians for the African & African American Ancestry. NA, not applicable.

^**c**^This allele is coded as “A” in Zheng et al. [1].

^**d**^Significant heterogeneity in risk estimates for different ancestries, see Table S3.

We then examined how the SNPs in the European 6–7% MAF classes were correlated in Yorubas, using the HapMap data. In YRI, the SNPs split into five separate equivalence classes, with MAFs ranging from about 6% (for the class tagged by rs9397435) to 1% (for the class containing only rs12665607)(Figure 2B). We typed these five SNPs in a sample of 851 breast cancer patients and 781 controls from Ibadan, Nigeria. We also included the key SNP rs2046210 and rs6929137, the V604I coding variant that is closely correlated with rs2046210 in Chinese and Europeans but not in Yorubas (Figure 2B). To confirm the associations observed in the Nigerians, we also typed the SNPs in a small set of African American breast cancer patients and controls from the Chicago area. Combined results from the two sample sets are shown in Table 1 and data from each of the two sets separately are shown in Table S2. Even though they are in different equivalence classes in Africans, nominally significant ORs were observed for rs9397435, rs12662670, rs12665607, and rs9383589 (Table 1). Inspection of the results from the Nigerians separately (Table 2, Table S2) and of the LD patterns in the data from the Nigerians and African Americans (Figure S3) did not suggest that the lack of resolution between these SNPs could be explained by European admixture in the African American samples. Neither the key SNP rs2046210 nor the coding variant rs6929137 showed any association with breast cancer risk in the African ancestry samples, hence they are unlikely to be causative or closely correlated with the causative variant. This is in agreement with Zheng et al. who previously reported that they were unable to see an association between rs2046210 or rs6929137 and risk in a sample of 810 African American breast cancer cases and 1,784 controls [9].

**Table 2:** Conditional analysis of rs9397435 association with breast cancer in European, Nigerian, and Taiwanese population samples.

Population Sample	Primary SNP	Allele	Frequency in Controls	Unadjusted		Adjusted for SNP	Adjusted	
				OR	*P*		OR*_adj_*	*P_residual_*
Combined European	rs9397435	G	0.063	1.15	1.2×10^−3^	rs9383932	1.16	4.2×10^−3^
"	rs9397435	G	"	"	"	rs12662670	1.14	0.019
"	rs9397435	G	"	"	"	rs12665607	1.08	0.51
"	rs9397435	G	"	"	"	rs9383589	0.99	0.95
"	rs9397435	G	"	"	"	rs3734805	1.11	0.34
"	rs9397435	G	"	"	"	rs6929137	1.14	3.9×10^−3^
"	rs9397435	G	"	"	"	rs2046210	1.14	6.1×10^−3^
"	rs9383932	G	0.132	1.06	0.066	rs9397435	1.00	0.91
"	rs12662670	G	0.071	1.12	6.4×10^−3^	rs9397435	1.00	0.96
"	rs12665607	A	0.072	1.14	1.1×10^−3^	rs9397435	1.04	0.69
"	rs9383589	G	0.070	1.15	6.2×10^−4^	rs9397435	1.13	0.22
"	rs3734805	C	0.072	1.13	1.8×10^−3^	rs9397435	1.02	0.87
"	rs6929137	A	0.316	1.04	0.082	rs9397435	1.01	0.58
"	rs2046210	T	0.337	1.04	0.099	rs9397435	1.02	0.41
Nigerian	rs9397435	G	0.063	1.39	0.016	rs12662670	1.34	0.047
"	rs9397435	G	"	"	"	rs12665607	1.32	0.068
"	rs9397435	G	"	"	"	rs9383589	1.31	0.11
"	rs9397435	G	"	"	"	rs3734805	1.43	0.049
"	rs9397435	G	"	"	"	rs6929137	1.39	0.019
"	rs9397435	G	"	"	"	rs2046210	1.44	0.047
"	rs12662670	G	0.027	1.36	0.16	rs9397435	1.17	0.51
"	rs12665607	A	0.010	1.76	0.079	rs9397435	1.33	0.43
"	rs9383589	G	0.016	1.66	0.045	rs9397435	1.31	0.37
"	rs3734805	C	0.028	1.33	0.16	rs9397435	0.94	0.82
"	rs6929137	A	0.538	0.97	0.67	rs9397435	0.95	0.56
"	rs2046210	T	0.716	1.00	0.96	rs9397435	0.91	0.73
Taiwanese	rs9397435	G	0.326	1.23	8.0×10^−4^	rs9383932	1.11	0.31
"	rs9397435	G	"	"	"	rs12662670	1.18	0.12
"	rs9397435	G	"	"	"	rs12665607	0.94	0.89
"	rs9397435	G	"	"	"	rs9383589	1.54	0.028
"	rs9397435	G	"	"	"	rs3734805	1.48	0.061
"	rs9397435	G	"	"	"	rs6929137	1.73	8.4×10^−4^
"	rs9397435	G	"	"	"	rs2046210	1.11	0.21
"	rs9383932	G	0.378	1.25	2.9×10^−4^	rs9397435	1.14	0.21
"	rs12662670	G	0.300	1.22	2.5×10^−3^	rs9397435	1.06	0.59
"	rs12665607	A	0.323	1.24	6.2×10^−4^	rs9397435	1.31	0.52
"	rs9383589	G	0.323	1.20	4.2×10^−3^	rs9397435	0.79	0.24
"	rs3734805	C	0.324	1.22	2.0×10^−3^	rs9397435	0.83	0.37
"	rs6929137	A	0.348	1.15	0.025	rs9397435	0.7	0.027
"	rs2046210	T	0.363	1.24	4.3×10^−4^	rs9397435	1.11	0.46

If a pathogenic variant is present in all three ancestries, then it might be expected to have a similar effect in all populations [8], [19]. A variant that is in strong LD with a pathogenic variant could also show similar properties, if the LD is maintained in different ancestral populations. Such variants are likely to show the strongest overall disease association when combined over all ancestries. In order to assess the genotyped variants for these characteristics, we used the Mantel-Haenszel model to obtain combined OR estimates and *P* values for the SNPs that had been typed in all three ancestral populations. The strongest breast cancer association overall, both in terms of OR and *P* value, was with rs9397435[G], a member of the European 6–7% MAF class, giving an OR of 1.19 and *P* = 3.90×10^−7^ (Table 1). The other four SNPs in the European 6–7% MAF class and 1–6% African MAF classes also showed substantial signals combined over all three ancestries. None of these five SNPs showed significant heterogeneity in OR estimates over the three ancestries (Table S3). However, all of the SNPs outside these classes (rs6929137 being an exception) showed significant heterogeneity between all three ancestries, or between Asians and Europeans, indicating that their effects are not consistent in all ancestries (Table 1, Table S3).

We then investigated whether the SNP with the strongest overall association could account for the signals observed in all three ancestries. In a multivariate analysis, no SNP in any ancestral group retained a significant at-risk signal when adjusted for the effect of rs9397435 (Table 2). Thus there is no evidence for an association signal in any of the ancestries that is not captured by rs9397435. In Europeans, rs9397435 retained significant ORs when adjusted for the effects of rs2046210, rs6929137, and marginally when adjusted for rs12662670 (Table 2). No significant OR*_adj_* was observed when rs9397435 was adjusted for rs12665607, rs9383589, or rs3734805 in Europeans. We take this to mean that, based on the available power, no tested SNP is more closely correlated with the causative variant in Europeans than rs9397435. However rs12665607, rs9383589, and rs3734805 are similarly correlated with the causative variant and cannot be distinguished from rs9397435 in this respect. Data from the Nigerians support the exclusion of rs2046210, rs6929137, and the tentative exclusion of rs12662670 from being the most strongly correlated with the causative variant but no additional resolution was achieved, the power again being limited by the sample size and low frequencies of the variants (Table 2). Data from the Taiwanese reconfirmed that little or no resolution is available within groups of Asian ancestry. We did note a significant protective effect of rs6929137[A] when adjusted for rs9397435 in Asians. This is most likely to be due to a fluctuation in the data since there is no sign of the effect in the other ancestries and there were no quality issues with the genotyping of rs6929137.

The pattern of risk associations was further illuminated by an examination of the common haplotypes generated by the typed SNPs (Table 3). In the Nigerians, the rs9397435[G] allele is present on several different, quite rare haplotypes (haplotypes E–I). All except one (haplotype G) have OR point estimates greater than 1. Two of these haplotypes (H and I) are more common in Europeans and Asians than in Nigerians, and are the dominant at-risk haplotypes in those population samples. Conversely, haplotypes E–G are vanishingly rare in Europeans and Asians. In Nigerians, the rs2046210[T] allele is present on all of the common haplotypes carrying rs9397435[G] (haplotypes E–I). However it is also present on two very common, non-risk haplotypes (A and B) and this effectively attenuates the association of rs2046210[T] with disease in Nigerians. In Europeans, haplotype B is lower in frequency but haplotype A is still present at substantial frequencies, again attenuating an association of rs2046210[T] with breast cancer. In Asians, haplotypes A and B are both very much lower in frequency while the at-risk haplotype H has become the dominant haplotype carrying rs2046210[T]. This illustrates how rs2046210[T] can be strongly associated with risk in Chinese, but only weakly if at all in the Europeans and Nigerians.

**Table 3:** Frequencies of common haplotypes in European, African, and Asian ancestry population samples.

		Nigeria		Iceland		U.S.A. European Ancestry		Taiwan	
Haplotype^a^	Haplotype ID	Frequency in Cases	Frequency in Controls	Frequency in Cases	Frequency in Controls	Frequency in Cases	Frequency in Controls	Frequency in Cases	Frequency in Controls
**TAAATTA**	A	0.346	0.368	0.225	0.232	0.239	0.233	0.012	0.018
**TGAATTA**	B	0.263	0.276	0.018	0.022	0.026	0.025	0.024	0.024
**TGAATCA**	C	0.164	0.158	0.673	0.679	0.635	0.646	0.550	0.590
**TAAATCA**	D	0.120	0.127	0.004	0.000	0.006	0.007	0.001	0.005
**TGAATTG**	E	0.028	0.015	0.000	0.000	0.002	0.000	0.001	0.000
**TAAATTG**	F	0.023	0.014	0.000	0.000	0.000	0.000	0.000	0.001
**TACATTG**	G	0.011	0.013	0.000	0.000	0.000	0.000	0.000	0.000
**GACGATG**	H	0.015	0.009	0.065	0.053	0.067	0.059	0.304	0.260
**TACGATG**	I	0.000	0.000	0.007	0.004	0.009	0.008	0.049	0.036
**GGAATCA**	J	0.000	0.000	0.006	0.004	0.004	0.007	0.023	0.023

^**a**^The SNPs and at-risk alleles are ordered as: rs12662670_G, rs6929137_A, rs3734805_C, rs9383589_G, rs12665607_A, rs2046210_T, rs9397435_G. Haplotypes with an observed control frequency of ≥0.01 in any one of the population samples are included.

To increase the resolution of the haplotype analysis, we generated a phylogenetic network based on HapMap data for 81 SNPs in the region (Figure S4). This confirmed that the haplotype group H forms a contiguous branch with much greater frequencies in Asians than in Africans. It also showed that the risk allele rs9397435[G] is present on a diversity of haplotype backgrounds in Africans, of which only some derivatives are represented in Europeans and Asians. Given the dispersion of the African haplotypes containing rs9397435[G], and assuming that all haplotypes carrying this allele indeed confer risk, there does not appear to be any HapMap SNP that could show a stronger association. We did note however that haplotype G, the only common haplotype for which we did not observe an OR point estimate greater than 1, is in an ancestral position in the group H branch of the network. This raises the possibility that the causative variant arose after the mutation event that created rs9397435.

We examined the genomic region containing the 6–7% European MAF class SNPs for correlations between SNP locations and known functional features. The SNPs occur in a region containing a number of ligand-inducible ER binding sites, suggesting that this area may be involved in autoregulation of the *ESR1* gene [20], [21]. However none of the SNPs in the 6–7% MAF group (including the ungenotyped ones) actually mapped within the identified ER binding sites. We noted that rs9397435 is located at a site of histone modification marks in human mammary epithelial cells (HMEC) and normal human keratinocytes (NHEK) that were experimentally verified by ChIP-Seq methodology [22]. Peaks of H3K4me1 and H3K4me2 (but not H3K4me3) co-localized with rs9397435. A moderate peak of H3K9ac was also seen at this location in HMEC. This pattern of histone modification has been associated with transcriptional enhancers but not with transcription initiation sites [23]. None of the other HapMap SNPs in the 6–7% MAF group showed similar associations with histone modification peaks or any other notable bioinformatic features.

To search for additional candidate causative variants, we accessed the April 2009 release of the 1000 Genomes project that includes data on 57 European individuals, 56 Yorubas and 59 Japanese or Han Chinese. We then looked for non-HapMap SNPs that are well correlated (r^2^≥0.75) with rs9397435 in both Europeans and Asians (no SNP was this highly correlated with rs9397435 in Yorubas). We identified 10 non-HapMap SNPs having this property of which 8 were listed in dbSNP build 130 and 2 were novel (Table S4). This list may not be exhaustive because the data originate from a draft release from the 1000 Genomes project. Nevertheless, the SNPs that were identified must be considered as potential causative variants. We searched for correlations between these 10 additional SNPs and locations of known functional features. A previously unknown C/T SNP at position 152,010,891 was seen to coincide with a ChIP-SEQ verified binding site of the transcriptional insulator protein CTCF in a variety of cell types including HMEC [24]. The variant changes a CpG sequence to TpG, the latter being correlated (r^2^ = 1 in CEU) with the rs9397435[G] breast cancer risk allele. Because CTCF binding is sensitive to cytosine methylation of CpG sites, we investigated the novel C/T SNP at 152,010,891 in more detail. We confirmed its existence by Sanger sequencing a sample of Europeans and generated a single-track Centaurus assay for it. The SNP is now listed as rs77275268 in dbSNP build 131. We confirmed its LD relations with rs9397435 in samples of European and Chinese ancestry (Table S5). We also found that rs77275268 exists in Africans at a MAF of 1.3% (in controls) and is most closely correlated there with rs9383589 among the typed HapMap SNPs (Table S5). Like rs9383589, it showed a nominally significant association with breast cancer in the African ancestry samples (OR = 1.97, *P* = 7.4×10^−3^). Bisulfite sequencing of peripheral blood DNA from 29 European individuals who were CC homozygotes for rs77275268 showed that the CpG sequence is partially methylated (Figure S5). The occurrence of the TpG variant at this position thus precludes facultative methylation and may affect CTCF binding.

To investigate a possible impact of the risk variants on gene expression, levels of *ESR1*, progesterone receptor (*PGR*) and HER2 (*ERBB2*) mRNAs were assessed in 1,234 frozen tumour samples (see *Materials and Methods*). SNP rs9397435 was genotyped using DNA samples from the same tumours. The at-risk GG homozygotes expressed mean levels of *ESR1* and *PGR* mRNA that were four to five-fold higher than the levels in AA homozygotes and AG heterozygotes (Figure S6). When assessed under our default, multiplicative (co-dominant) inheritance model, these differences were of borderline significance for *PGR* and not significant for *ESR1* (Table S6). Assessed under a recessive inheritance model, the increases in both *ESR1* and *PGR* mRNA levels in GG homozygotes were significant (*P* = 0.024 and 0.031 for *ESR1* and *PGR* respectively, (Table S6)). In comparisons with the full genotype model, neither the multiplicative nor the recessive models could be rejected. *ERBB2* mRNA levels did not vary with genotype. These findings raise the possibility that rs9397435[G] (or a correlated SNP) might act to increase expression of *ESR1* and, as a consequence, increase *PGR* expression. However, we caution that these findings should be considered only as hypothesis-generating since the GG homozygotes are rare and the significance is marginal. Moreover, we saw no evidence that the association between rs9397435[G] and breast cancer risk *per se* showed a recessive pattern of inheritance (*P* = 0.75 and 0.93 for a test of the multiplicative vs the full genotype model in Europeans and Taiwanese respectively).

We reviewed the medical records of approximately 8,441 European and Taiwanese patients, including 1,792 from a series of case-only cohorts. The rs9397435[G] allele was found to confer significant risk of both ER positive and ER negative breast cancer and of both progesterone receptor positive and negative disease (Table S7, Table S8). These results were puzzling in light of the proximity of the risk variant to the *ESR1* gene and the putative effect of rs9397435[G] homozygosity on gene expression described above. However the observed association with both ER positive and ER negative disease is in agreement with Zheng et al., who reported significant associations with both ER positive and ER negative breast cancer in Chinese women, with a higher OR for ER negative breast cancer than for ER positive disease [1]. Clearly, the phenotypic effect of the risk variant merits further investigation. In Europeans, we observed that rs9397435[G] was associated at nominal significance with an earlier age at first invasive breast cancer (*P* = 0.015, Table S7), although this effect was not evident in the Taiwanese patient sample (Table S8).

## Discussion

In summary, we have shown that the initially reported [1] Chinese breast cancer risk variant rs2046210 cannot be effectively used as a risk marker in Europeans and Africans because it does not tag the causative variant(s) in all three ancestries. We have identified a variant, rs9397435[G], that confers risk of breast cancer with a consistent effect in all three main ancestral populations and that can fully explain the association signal in each population. The frequency of the rs9397435[G] risk allele is substantially lower in Europeans and Africans (6.3% in controls) than it is in Taiwanese (32.6%). This limits the power to detect an effect of this variant with confidence even in a large sample of Europeans as was used here. It may also explain why the variant was not detected in previous genome-wide association studies conducted in Europeans [4], [17], even though the class was reasonably well tagged on the Illumina chips used (Figure 2).

In addition to rs9397435, three other HapMap SNPs rs12665607, rs9383589, and rs3734805 are consistently associated with risk in all three ancestries and cannot be distinguished from rs9397435 based on the currently available data. This highlights the fact that, in general, the resolution of fine mapping is very sensitive to power restrictions. For example, inspection of the haplotype distribution (Table 3) suggests that, given a sufficiently large sample of Chinese, it might be possible to resolve haplotypes A and B from haplotypes H and I (thereby resolving rs2046210 from rs9397435) in this ancestral group alone. As we have shown, shifting the analysis between ancestries can provide some additional resolution without resorting to very large sample sizes. It can also be seen (Figure 2B) that the greatest potential resolution is offered by Africans, populations where the collection of large samples is challenging. Naturally, the ancestry shift approach is only viable if the variant arose before population divergence and even if the variant is present in multiple ancestral populations, the optimum strategy can vary from locus to locus [25].

Haplotype analysis conducted by [1] indicated that rs2046210[T] is present on multiple at-risk haplotypes, consistent with the presence of a single, common causative variant highly correlated with rs2046210 in Chinese. However an observed association with a common SNP can also arise from a set of multiple underlying pathogenic variants [26]. In this study we do not make the presumption of a single underlying causative variant, although for simplicity we refer to a single variant. Under the single variant hypothesis, refinement mapping will identify SNPs closely correlated with the causative variant and the causative variant itself is expected to be one of those variants which give the strongest association signals. Under a multiple pathogenic variant hypothesis, refinement mapping will identify SNPs that tag the set better (although one might expect it to be more difficult to identify SNPs that show homogeneity of effect between ancestral groups). However in this case the group of tagging SNPs giving the strongest signals will not necessarily contain pathogenic variants. For some practical purposes, such as genetic risk assessment, distinction between the two hypotheses may not be important, but the difference is crucial if the aim is to identify the underlying pathogenic genetic lesion(s). An example is seen in the LOXL1 locus where two nonsynonymous SNPs, thought to be pathogenic variants, account for an association with exfoliation glaucoma. The strongest association in the GWAS arose from a third SNP (rs2165241) that is in LD with both nonsynonymous variants [27]. In this study we have presented evidence that both rs9397435 and rs77275268 are located at sites of potential functional significance. While these SNPs merit further investigation, we note that in the absence of conclusive evidence of a single underlying causative variant, the search for pathogenic variants need not be restricted to SNPs highly correlated with rs9397435 in Asians and Europeans.

## Materials and Methods

### Ethics statement

This work was approved by the National Bioethics Committee of Iceland and the Icelandic Data Protection Authority and by the respective local review boards for the samples provided by external collaborators.

### Samples

The breast cancer case∶control population samples are listed and referenced in Table S1. The Netherlands (Rotterdam) case-only DNA samples were isolated from 1,792 frozen tumour specimens. Only primary tumours were used and none of the patients had received neo-adjuvant treatment. The year of surgery was between 1978 and 2002. The patients' ages ranged from 22 through 88 years at diagnosis. 1748 patients were M0 at diagnosis, while 44 showed metastatic disease (M1). 1100 patients had lymph node negative disease, 676 were diagnosed with involved lymph nodes and for 16 patients this information was missing. ER was determined in 1783 tumours, 453 were negative (<10 fmol/mg protein) and 1330 were positive. Further details of these patient cohorts have been published previously [28], [29].

The U.S.A. (Chicago) samples were from individuals of self-reported African American ancestry, drawn from the Chicago Cancer Prone Study (CCPS), which is an ongoing hospital-based case∶control investigation designed to study the genetics of breast cancer in young patients. Cases with histologically confirmed breast cancer were enrolled through the Cancer Risk Clinic at the University of Chicago. Early-onset cases and African Americans were oversampled. Controls without breast cancer were gender and age-matched with cases and enrolled from patients who visit the same hospital and are wiling to donate blood for genetic studies. Similar to the Nigerian Breast Cancer Study, the CCPS adopted the questionnaire of the Breast Cancer Family Registry. Blood samples were collected from cases and controls and used for DNA isolation. Pathological and clinical data were collected for cases. The study is associated with the University of Chicago Specialized Program of Research Excellence (SPORE) programme.

### Genotyping

Genotyping was carried out using Nanogen Centaurus assays [18]. Assays were validated by genotyping on HapMap CEU, YRI and CHB/JPT samples and comparing the genotypes with the published data. Assays were rejected if they showed ≥1.5% mismatches with the HapMap data. Genotyping of Icelandic and foreign samples was carried out at the deCODE Genetics facility. Clustering algorithms were applied and manual editing was carried out in a standardized manner for all sample sets. Two standard control DNA samples and water blanks were included on every plate. Genotyping yields were in excess of 98% for all SNP-Sample combinations attempted.

#### Bisulfite sequencing

Bisulfite treatment of 1 µg of each peripheral blood DNA sample was conducted with EpiTect Bisulfite Kit (QIAGEN-59104) according to the manufacturer's protocol. PCR and Sanger sequencing was conducted with standard protocols.

### Gene expression analysis

RNA isolation and quantitative RT-PCR analysis was carried out as described previously [30]. Briefly, tumour material was preserved in liquid nitrogen and RNA isolated from 20–60 cryostat sections of 30µm using standard methods. cDNA was synthesized using oligo (dT) and random hexamer primers. Real-time quantitative PCR was done on an ABI Prism 7700 Sequence Detection System (Applied Biosystems) using assay primers described in [30], [31]. Ct values for the target genes were normalized to the mean Ct values of three housekeeping genes (*HMBS*, *HPRT* and *B2M*) and expressed as: Relative Expression Level = 2^(mean Ct housekeeping−mean Ct target)^.

### Statistical analysis

To construct dendrograms, SNPs were arranged in hierarchical clusters based on the r^2^ relationships between them. The clustering was performed using the “stats” package of R software. The “hclust” command was used with the method “average”. Pairwise r^2^ values were first re-arranged into a bi-dimensional matrix M that was transformed into a similarity matrix by performing the operation 1-M. The similarity matrix obtained was finally used as a distance matrix and depicted by a dendrogram. In this similarity matrix an original r^2^ value of 1 is thus transformed to 0, representing a distance of 0 from a fully correlated SNP.

We calculated the OR for each SNP allele assuming the multiplicative model, *i.e.* assuming that the relative risk to a person is the product of the relative risks of each of the two alleles carried. This assumption was tested by calculating genotype-specific relative risks for each SNP in Europeans and comparing them to those determined under the multiplicative model. No significant deviations from the multiplicative model were observed. Therefore, allelic OR and *P* values are presented in the data tables. *P* values were calculated with the standard likelihood ratio χ^2^ statistic and confidence intervals were calculated assuming that the estimate of OR has a log-normal distribution. Some Icelandic cases and controls are related to each other, causing the χ^2^ statistic to have a mean >1. We estimated the inflation factor using a previously described procedure in which genotypes were simulated through the genealogy of 731,175 Icelanders and the χ^2^ corrected statistics accordingly [32]. The inflation factor for the set of Icelandic samples used in this study was 1.08 and all *P* values cited have been adjusted accordingly. Individuals in the replication sample sets were assumed to be unrelated to each other. In this study we did not carry out a comprehensive scan of the region in Europeans. Rather, we tested a specific hypothesis; i.e. whether any of the European equivalence classes that correspond to the Asian equivalence class of rs2046210, confer risk of breast cancer in Europeans. The appropriate multiple testing adjustment therefore takes into consideration the number of equivalence classes tested in each ancestry.

The tested SNPs are in LD with each other. Therefore, wherever the genotype of one SNP was missing for an individual, the genotypes of the correlated SNPs were used to infer the missing genotypes using a likelihood approach as described previously [33]. This ensured that the same set of individuals is tested for each SNP. Thus, all *P* values are based on the same individuals, making comparisons more straightforward. Joint analyses of multiple case∶control replication groups was carried out using a Mantel-Haenszel model in which the groups are allowed to have different population allele frequencies but were assumed to have common relative risks. This was done when combining the various European sample sets, when combining the Nigerians and African Americans, and when combining samples of different ancestries. Tests of heterogeneity were performed by comparing the null hypothesis of the effect being the same in all populations to the alternative hypothesis of each population having a different effect using a likelihood ratio test. *I* ^2^ takes values between 0% and 100% and describes the proportion of the total variation in estimates that is due to heterogeneity [34].

Haplotype frequencies using the genotyped SNPs were estimated by maximum likelihood using the haplotype analysis program NEMO [35]. For the haplotype analysis using HapMap data, phased haplotypes were generated for the 60 CEU parents, 60 YRI parents and 90 Asian individuals. The phases of alleles in haplotypes was estimated using the EM algorithm, in combination with the family trio information for the CEU and YRI groups (where the genotypes from the 30 children were used to help infer the allelic phase of the haplotypes).

Quantitative RT-PCR data were analyzed under the multiplicative model by regressing log_10_ transformed Relative Expression Level values against the number of risk alleles carried (0,1,2). When testing the recessive model, we used the GG homozygote status as an explanatory variable taking values 0 (AA homozygote or AG heterozygote) or 1 (GG homozygote). In the full model, we used two explanatory variables; the AG heterozygote status (0 or 1) and the GG homozygote status (0 or 1).

### Bioinformatics

For the list of candidate SNPs we carried out a search for overlaps between SNP position and known bioinformatic features. We retrieved data from the UCSC human genome browser and from the UCSC test browser (HG18 build 36) [36]. We also retrieved data from three bioinformatic feature publications [20], [21], [37]. We accessed all available feature tracks containing genome positional information (approximately 10,000 tracks) and identified those features that overlapped with SNPs. Data from the 1000 Genomes project were obtained from the April 2009 release (http://www.1000genomes.org). These are recognized draft quality data and were used as is without quality filtering. All genomic locations quoted are from HG18 build 36.

## Supporting Information

Figure S1 Overview of LD at the *C6orf97-ESR1* locus. The figure shows a view of the genomic region of chromosome 6, nucleotides 151,930,000–152,200,000 taken from the UCSC browser Build 36 assembly (hg18). The track “Eq class SNPs” shows the locations of the SNPs that are correlated (r^2^>0.65) with rs2046210 in Han Chinese. The *C6orf97* and the four RefSeq isoforms of *ESR1* are shown. Below that are LD plots of D′ (a) and r^2^ (b) between pairs of SNPs in the region. Darker shading indicates stronger LD values. Data are based on phased genotypes from HapMap Phase II release 22.
(0.22 MB DOC)

Figure S2 Dendrogram showing r^2^ relationships between *C6orf97-ESR1* SNPs in HapMap Han Chinese (CHB). On the left are listed the SNPs that are correlated with an r^2^>0.65 with rs2046210. The SNPs are arranged in a hierarchical cluster dendrogram based on the r^2^ values between them derived from the HapMap Phase II release 23a genotypes. Note that the scale on the top of the panel shows 1-r^2^ values (*i.e.* a value of 0 corresponds to an r^2^ of 1). The scale is limited in range because the SNPs were selected to have r^2^ values greater than 0.65.
(0.91 MB DOC)

Figure S3 Dendrograms showing r^2^ relationships between the *C6orf9-ESR1* SNPs genotyped in each study population. On the left are listed the SNPs that were genotyped in each of the study population samples. The name of the study population sample is indicated on the right of each panel. The SNPs are arranged in a hierarchical cluster dendrogram based on the r^2^ values between them derived from the observed genotypes for the SNPs. Note that the scales on the top of the panels show 1-r^2^ values (*i.e.* a value of 0 corresponds to an r^2^ of 1). The scale for the Taiwanese sample is limited in range between 0 and 0.4 (corresponding to an r^2^ range of 1 to 0.6) because all genotyped SNPs had r^2^ values greater than 0.6. The scale for the USA, African-American ancestry and the Nigerians ranges from 0.3 to 1.0 (corresponding to an r^2^ range of 0.7 to 0) because no pair of genotyped SNPs had r^2^ values between them of greater than 0.7.
(2.35 MB DOC)

Figure S4 Haplotype analysis of the *C6orf97-ESR1* region. Shown is a median joining (MJ) network describing the evolutionary relationships between haplotypes inferred from the genotypes of 81 HapMap (Phase II release 21) SNPs in the region chr6:151,950,821–151,992,990. Each haplotype is represented by a circle whose area reflects the overall number of copies observed and whose colour coding indicates the frequency of the haplotype in the different ancestral groups as indicated in the figure. Lines between the circles represent mutational evolutionary pathways between haplotypes reconstructed by the MJ algorithm. The line length is proportional to the number of inferred mutational differences between haplotypes. Black nodes represent non-sampled haplotypes that were reconstructed by the MJ algorithm as evolutionary intermediates between observed haplotypes. Encircled clusters of haplotypes are those carrying the rs9397435[G] allele and their lettering corresponds to the haplotype IDs shown in Table 3. Haplotypes K and L contain the rs9397435[G] allele but were too rare to qualify for inclusion in Table 3. Note that in Asians and Europeans, haplotypes bearing rs9397435[G] are clustered on the H, I and L branches, whereas in Yorubas the rs9397435[G] allele is found on more widely dispersed branches. Note also that Haplotype G, which was observed only in Yorubas, is in an ancestral position on the main H,I,L branch.
(0.14 MB DOC)

Figure S5 Bisulfite sequencing of region surrounding the C/T SNP at position 152,010,891 (arrowed) showing differential methylation of the C nucleotide in CC homozygotes. The top line shows the reference (non-bisulfite treated) sequence. Panels *a–d* show sequence traces of bisulfite-treated DNA from four CC homozygous individuals. In samples *a* and *b* the C nucleotide is predominantly methylated while a minority is unmethylated. In sample *c*, the C is predominantly unmethylated and in sample *d* similar amounts of methylated and unmethylated C are present. At neighboring C nucleotides, the conversion of unmethylated cytosine is complete, indicating that the bisulfite treatment was effective. In addition, we noted that nearby CpGs at positions 152,010,768, 152,010,842, 152,010,940, 152,011,003 and 152,011,013 were also methylated or partially methylated.
(0.43 MB DOC)

Figure S6 Quantitative RT-PCR analysis of *ESR1* (ER), *PGR* (PG) and *ERBB2* (HER2) mRNAs in tumours with different genotypes for rs9397435. RNA and DNA was isolated from 1,234 frozen tumour specimens. RNA levels were analyzed by RT-PCR and normalized to the mean level of three housekeeping genes. Relative expression levels are calculated as 2^(mean Ct housekeeping−mean Ct target)^. Genotypes of rs9397435 were determined by Centaurus assay. Numbers of individuals with each genotype are 1,072 (AA), 151 (AG) and 11 (GG). Histogram displays the mean relative expression level (calculated as 10^mean of log10 of relative expression level^) for each genotype. Error bars indicate the standard error of the mean relative expression levels.
(0.05 MB DOC)

Table S1 Overview of the sample sets used in the study.
(0.05 MB DOC)

Table S2 Association of *C6orf97/ESR1* SNPs with breast cancer in each population sample.
(0.20 MB DOC)

Table S3 Heterogeneity in risk estimates for combined population samples.
(0.06 MB DOC)

Table S4 Non-HapMap SNPs in strong LD with rs9397435 identified from 1,000 Genomes Project Data^a^.
(0.05 MB DOC)

Table S5 LD relations with the novel chr6:152,010,891[C/T] SNP (rs77275268).
(0.04 MB DOC)

Table S6 Levels of ERα, PR, and HER2 mRNA in primary tumours, stratified by rs9397435 genotype and assessed under multiplicative and recessive inheritance models.
(0.05 MB DOC)

Table S7 Stratification by clinical variables of breast cancer associations with rs9397435[G] in combined European ancestry population samples^a^.
(0.11 MB DOC)

Table S8 Stratification by clinical variables of breast cancer associations with rs9397435[G] in Taiwan^a^
(0.11 MB DOC)
