# Supplementary figures and images for: Ancestry-Shift Refinement Mapping of the C6orf97-ESR1 Breast Cancer Susceptibility Locus
Source: PLoS Genet. 2010 Jul 22;6(7):e1001029. doi: 10.1371/journal.pgen.1001029 (PMC2908678; doi:10.1371/journal.pgen.1001029)

**Figure S1a:**


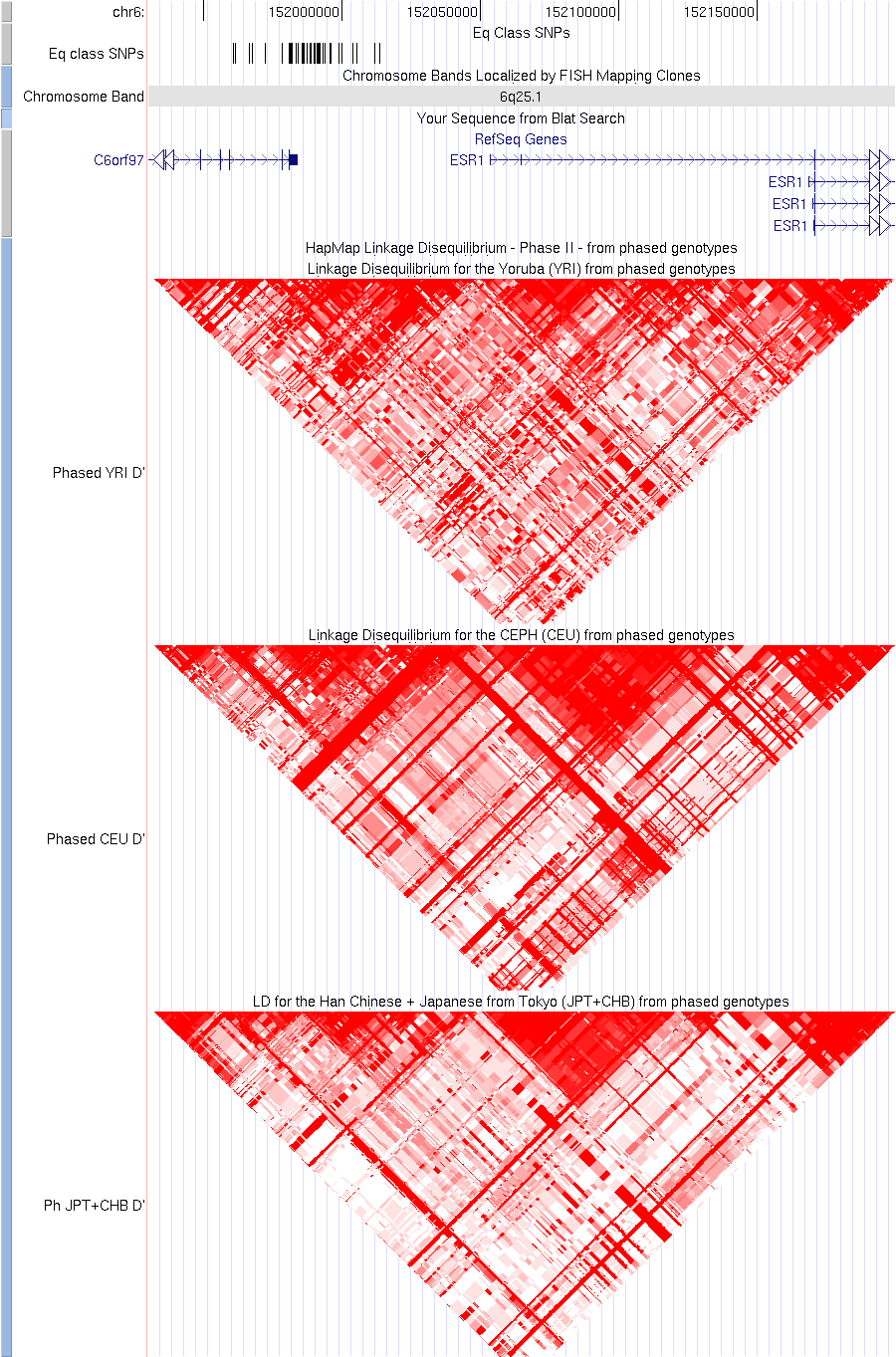


**Figure S1b:**


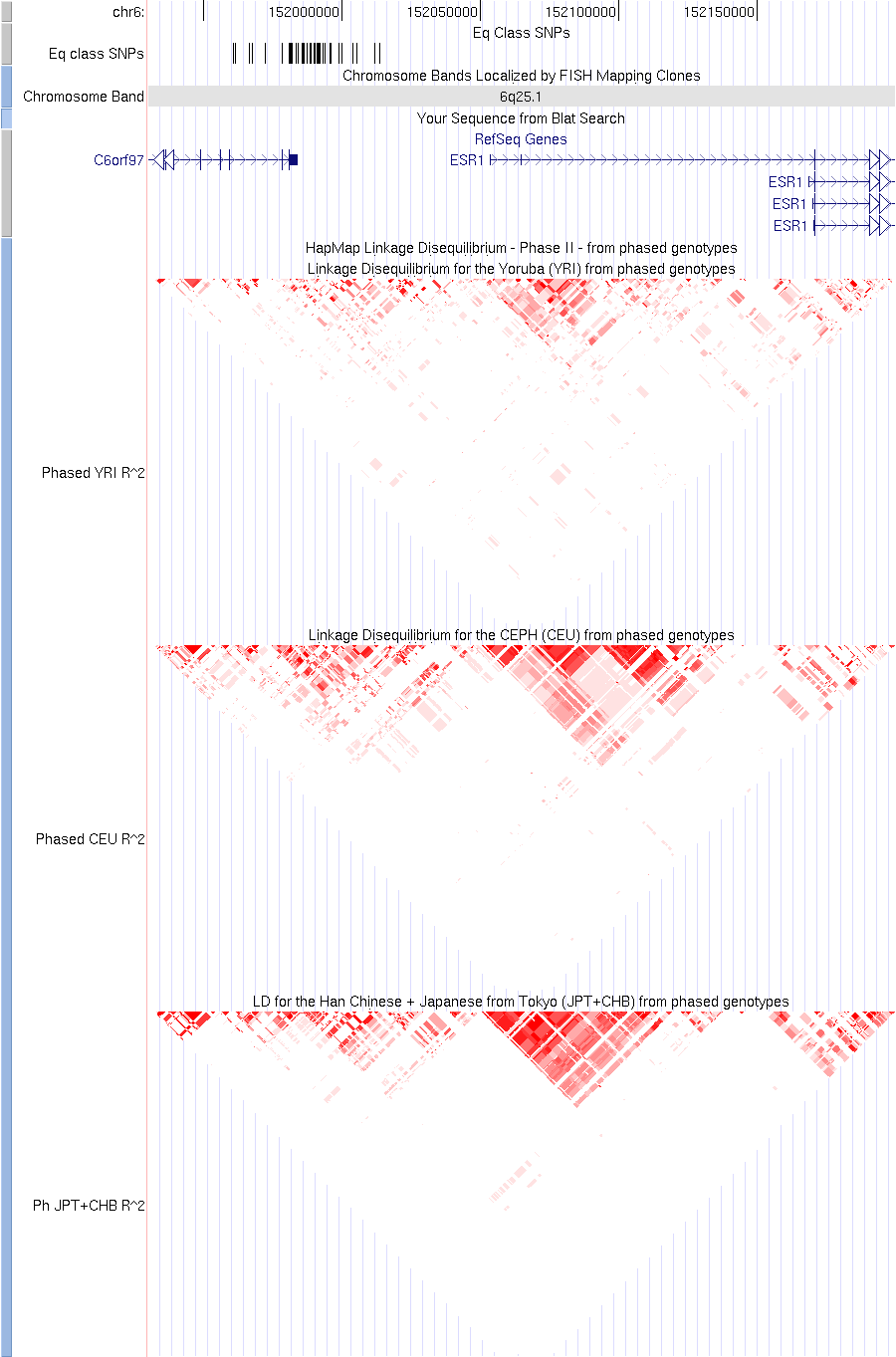

Supplement: Figure S1 — Overview of LD at the C6orf97-ESR1 locus. The figure shows a view of the genomic region of chromosome 6, nucleotides 151,930,000–152,200,000 taken from the UCSC browser Build 36 assembly (hg18). The track “Eq class SNPs” shows the locations of the SNPs that are correlated (r2>0.65) with rs2046210 in Han Chinese. The C6orf97 and the four RefSeq isoforms of ESR1 are shown. Below that are LD plots of D′ (a) and r2 (b) between pairs of SNPs in the region. Darker shading indicates stronger LD values. Data are based on phased genotypes from HapMap Phase II release 22. (0.22 MB DOC) [file pgen.1001029.s001.doc]

**Figure S2:**


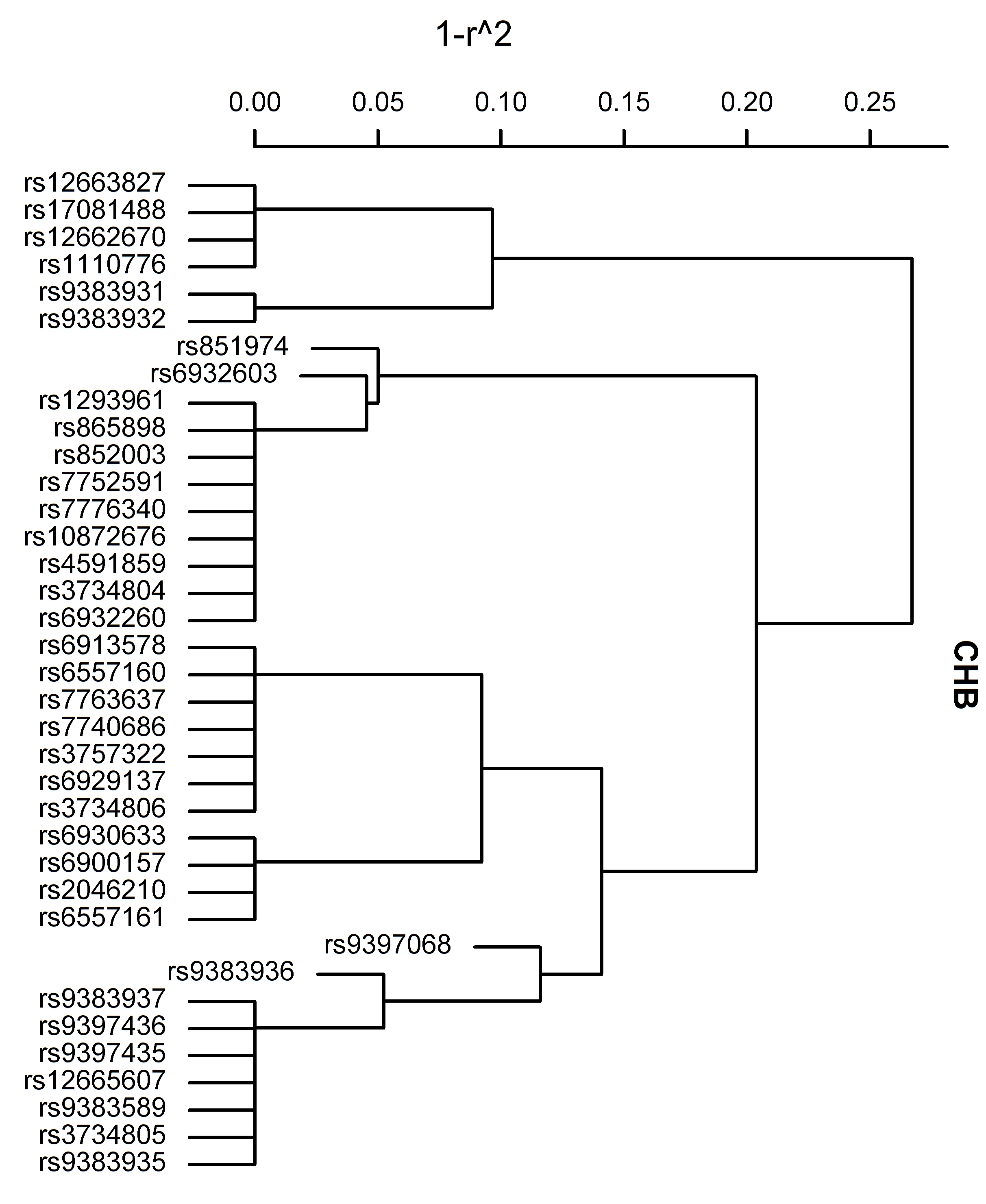

Supplement: Figure S2 — Dendrogram showing r2 relationships between C6orf97-ESR1 SNPs in HapMap Han Chinese (CHB). On the left are listed the SNPs that are correlated with an r2>0.65 with rs2046210. The SNPs are arranged in a hierarchical cluster dendrogram based on the r2 values between them derived from the HapMap Phase II release 23a genotypes. Note that the scale on the top of the panel shows 1-r2 values (i.e. a value of 0 corresponds to an r2 of 1). The scale is limited in range because the SNPs were selected to have r2 values greater than 0.65. (0.91 MB DOC) [file pgen.1001029.s002.doc]

**Figure S3:**

**
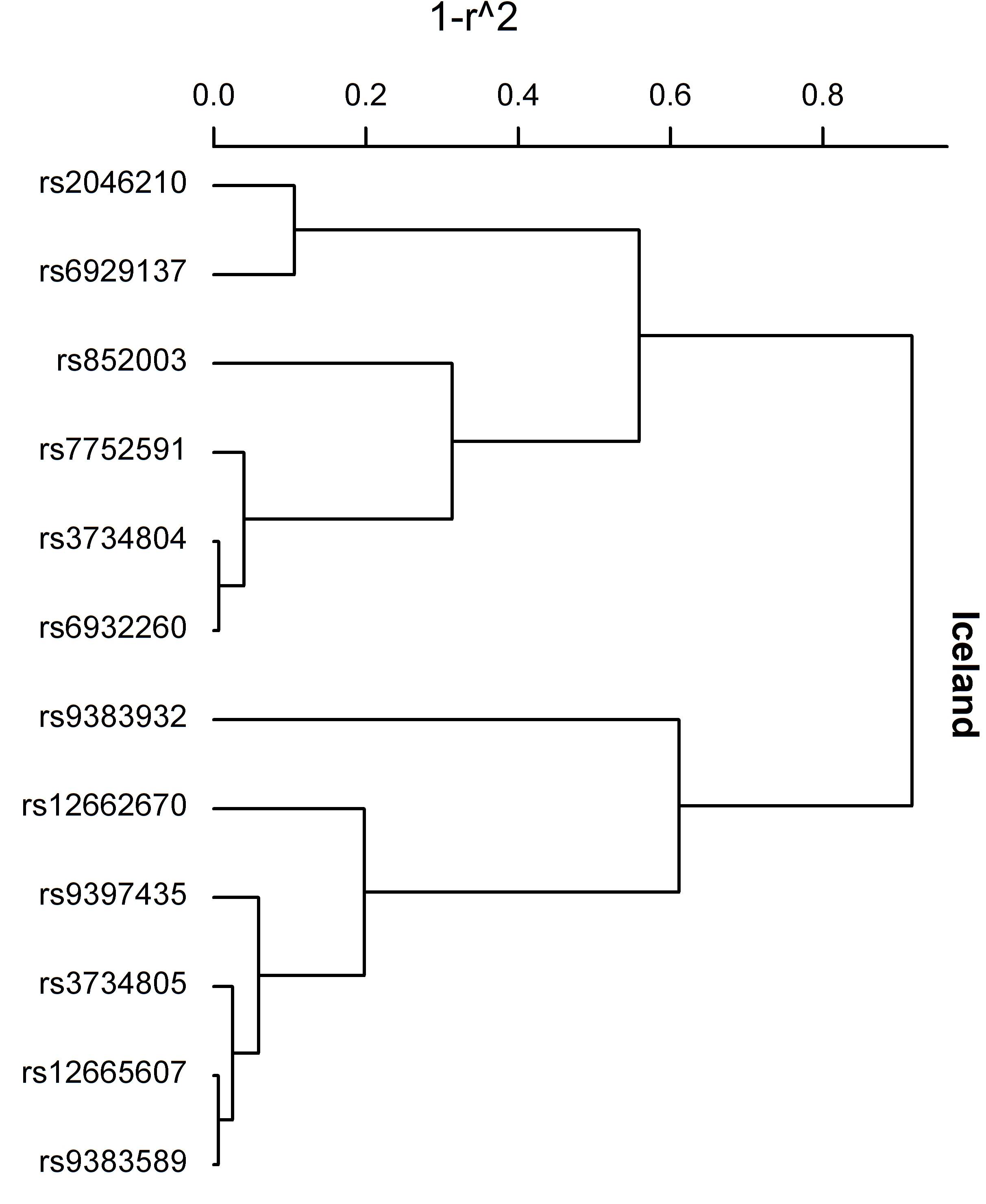
**


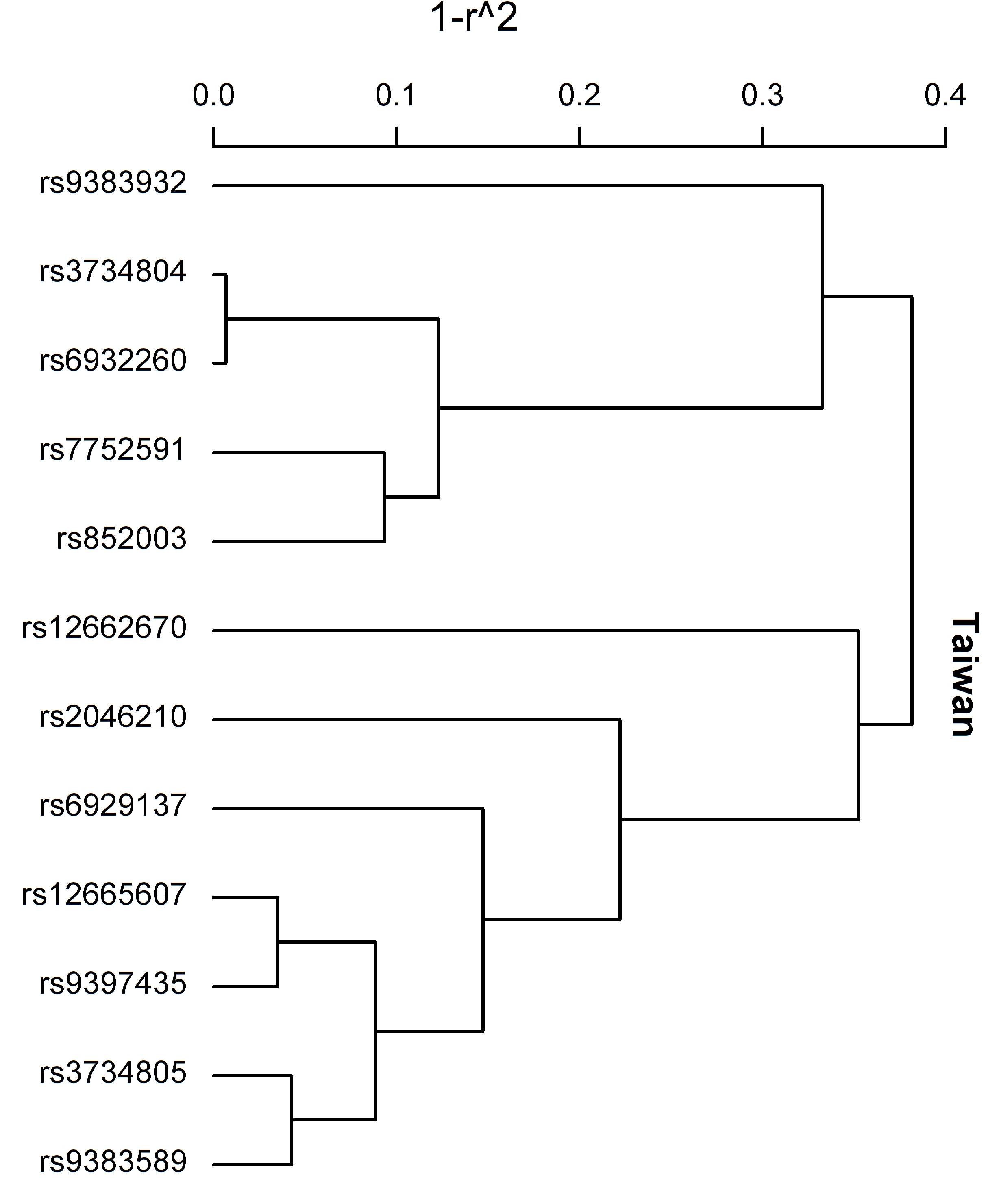


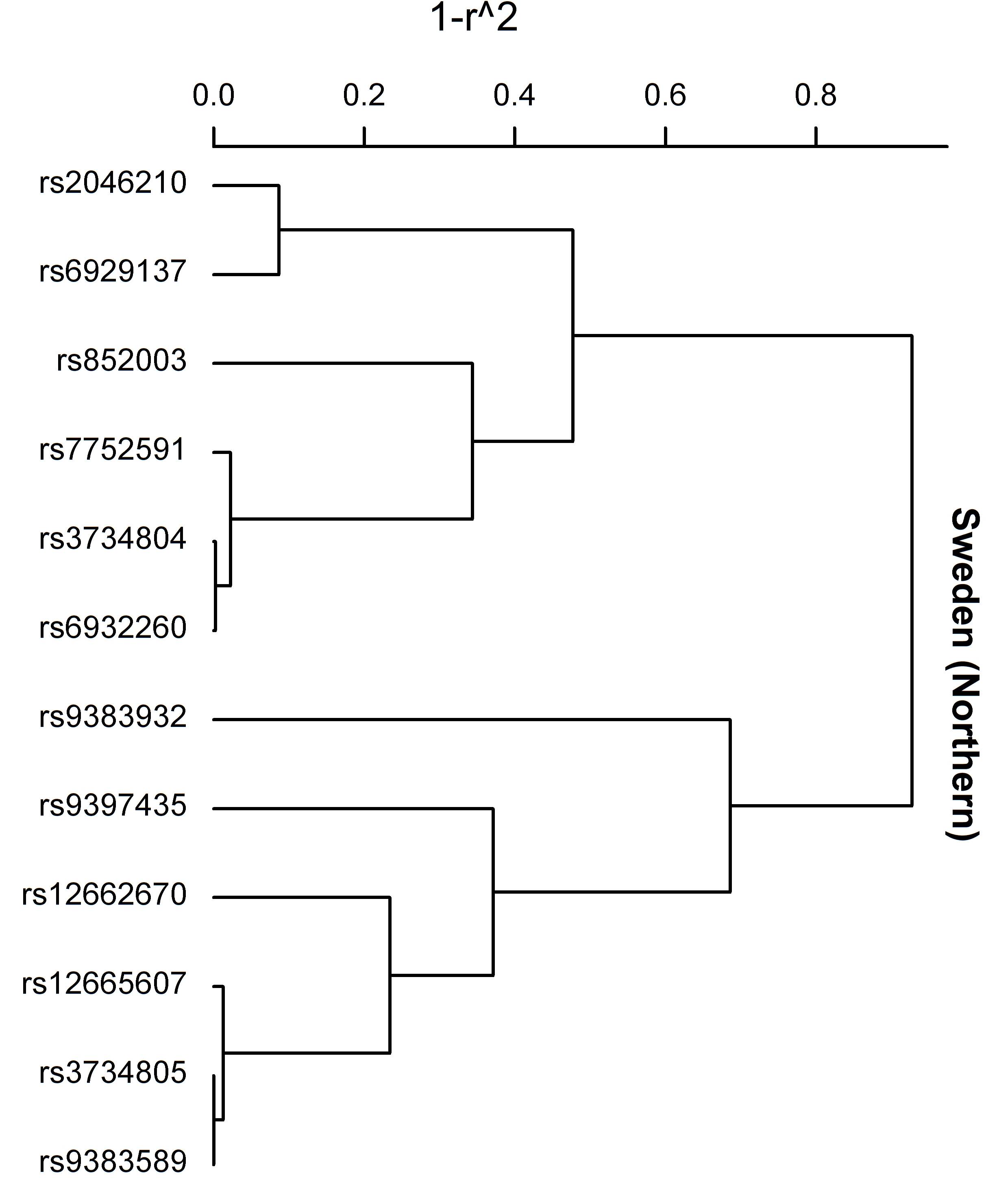

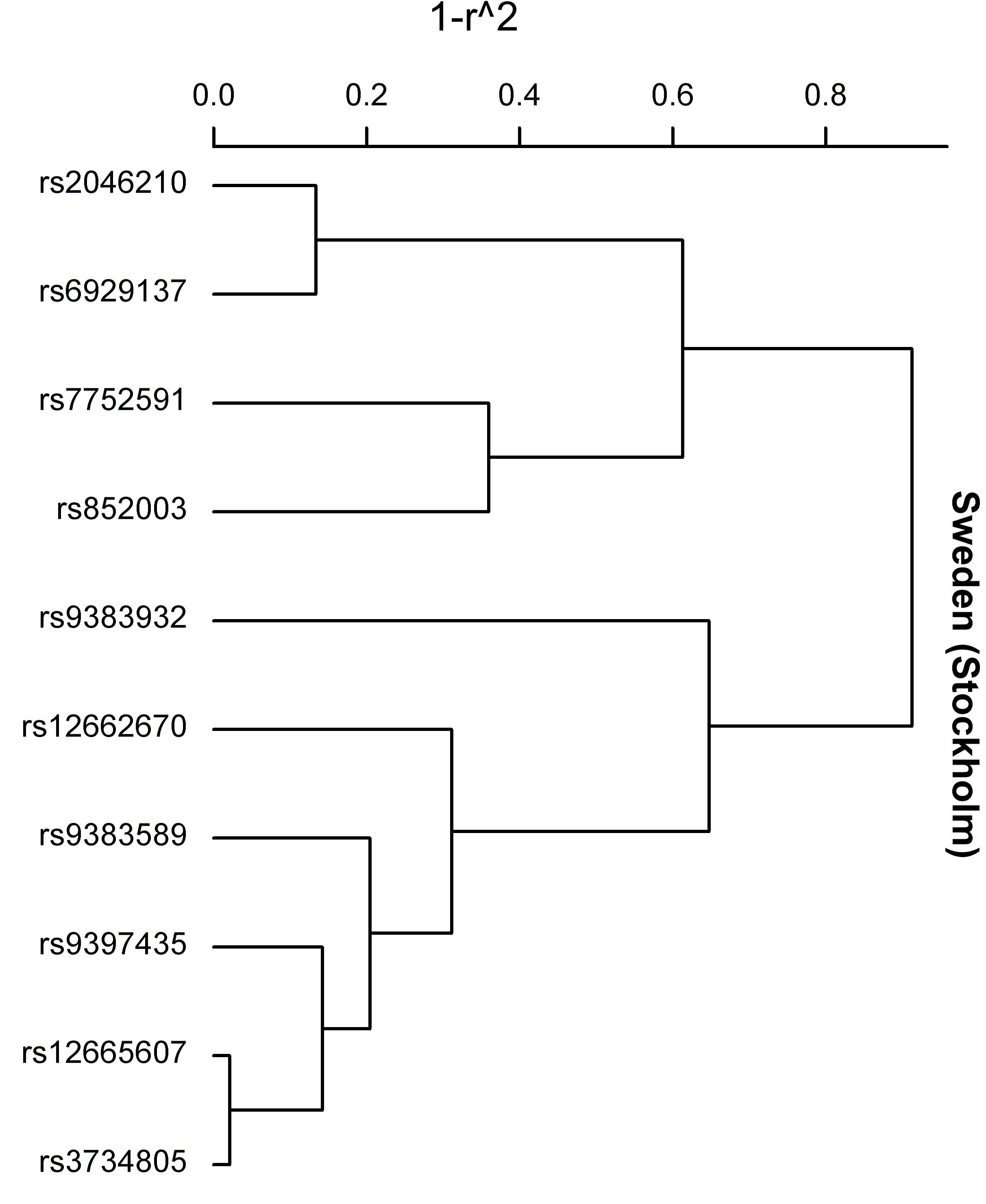


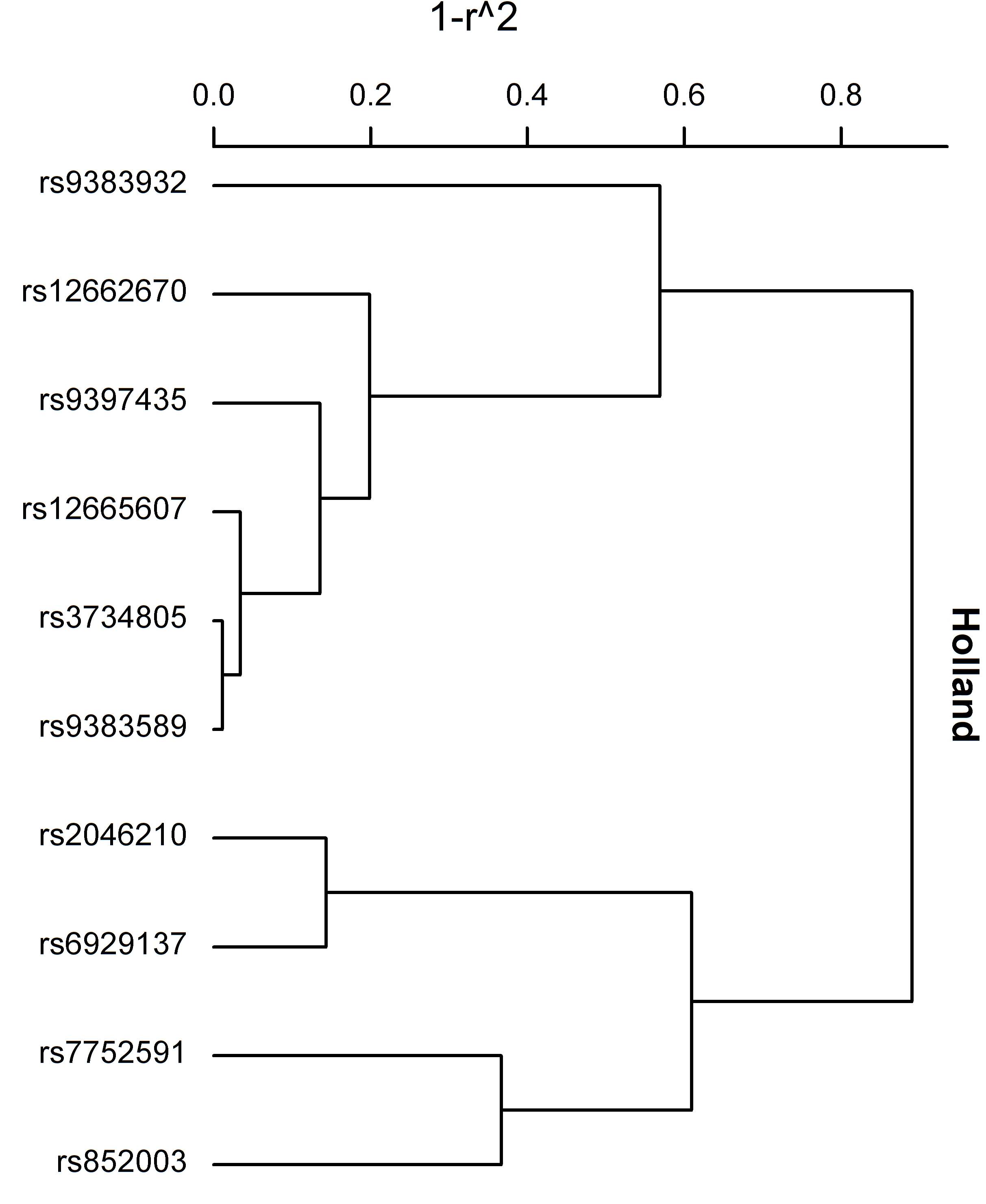

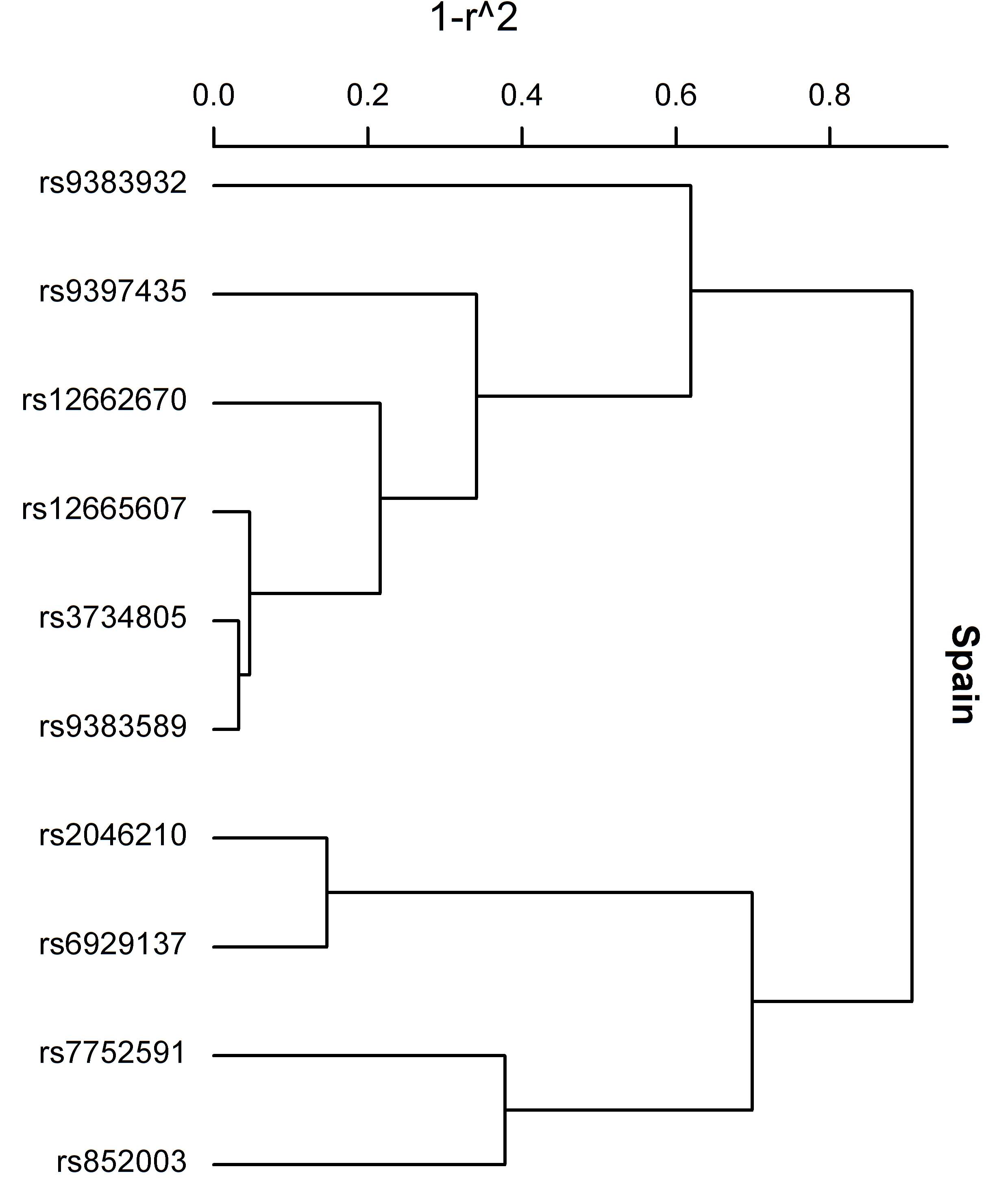


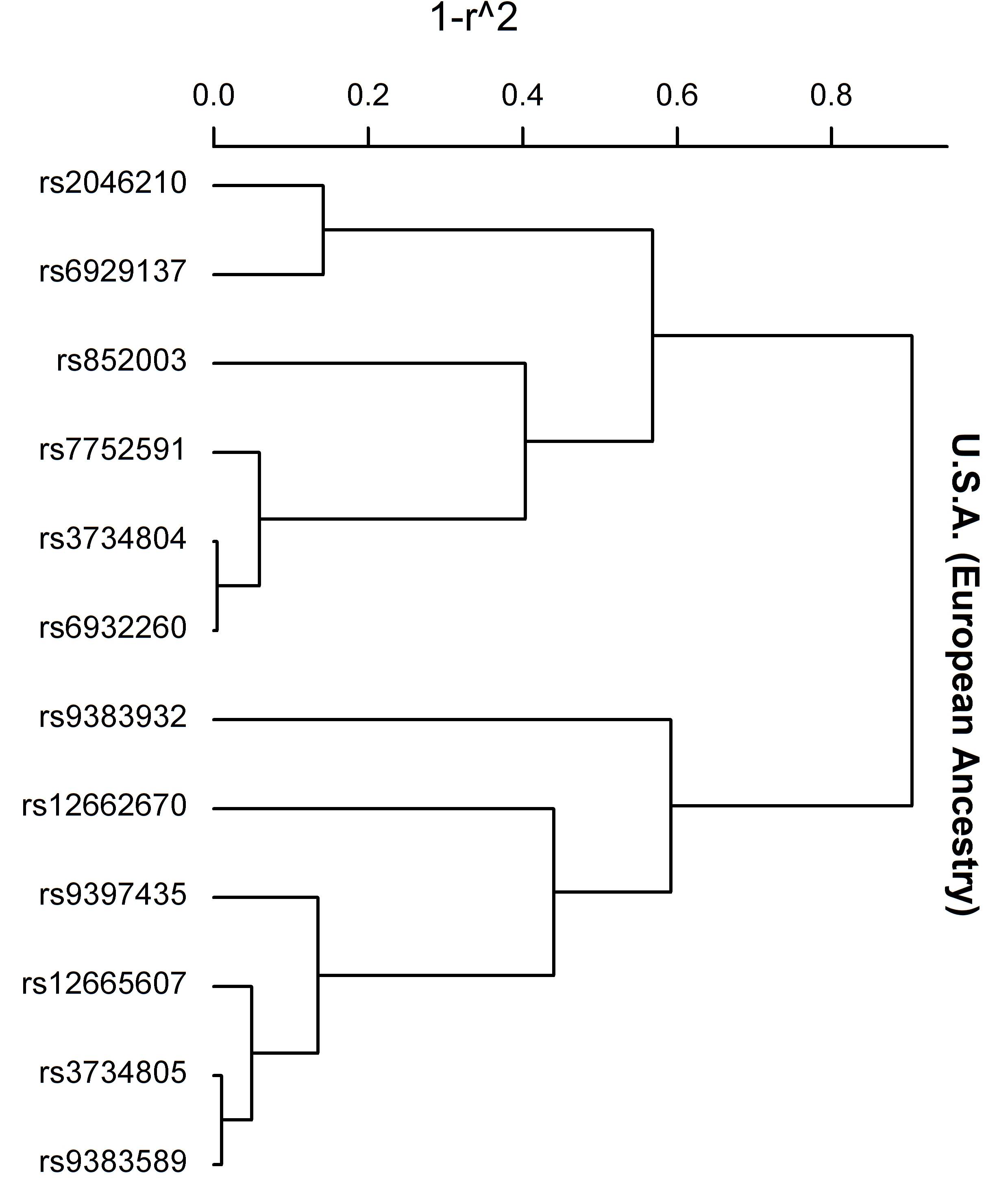


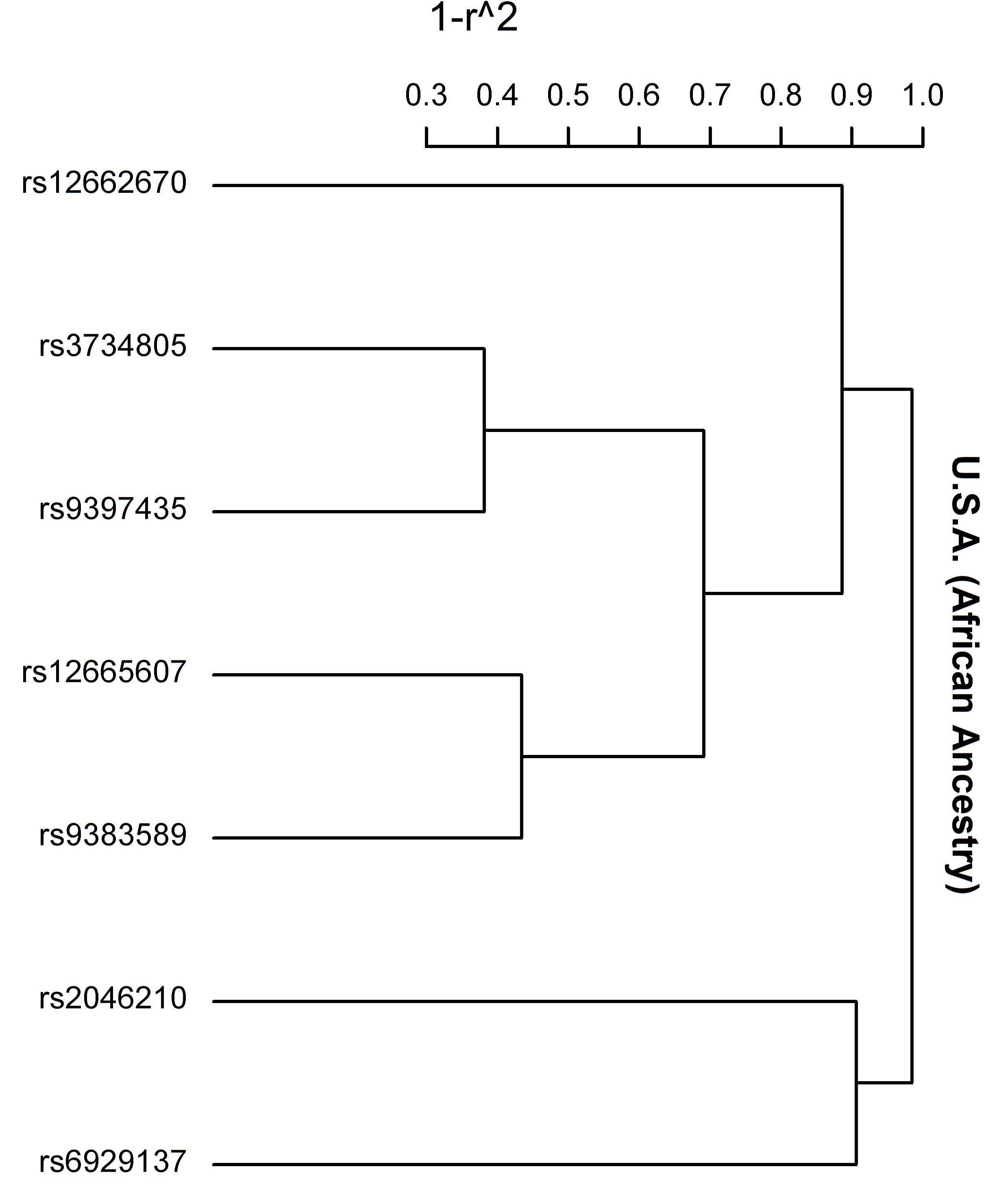

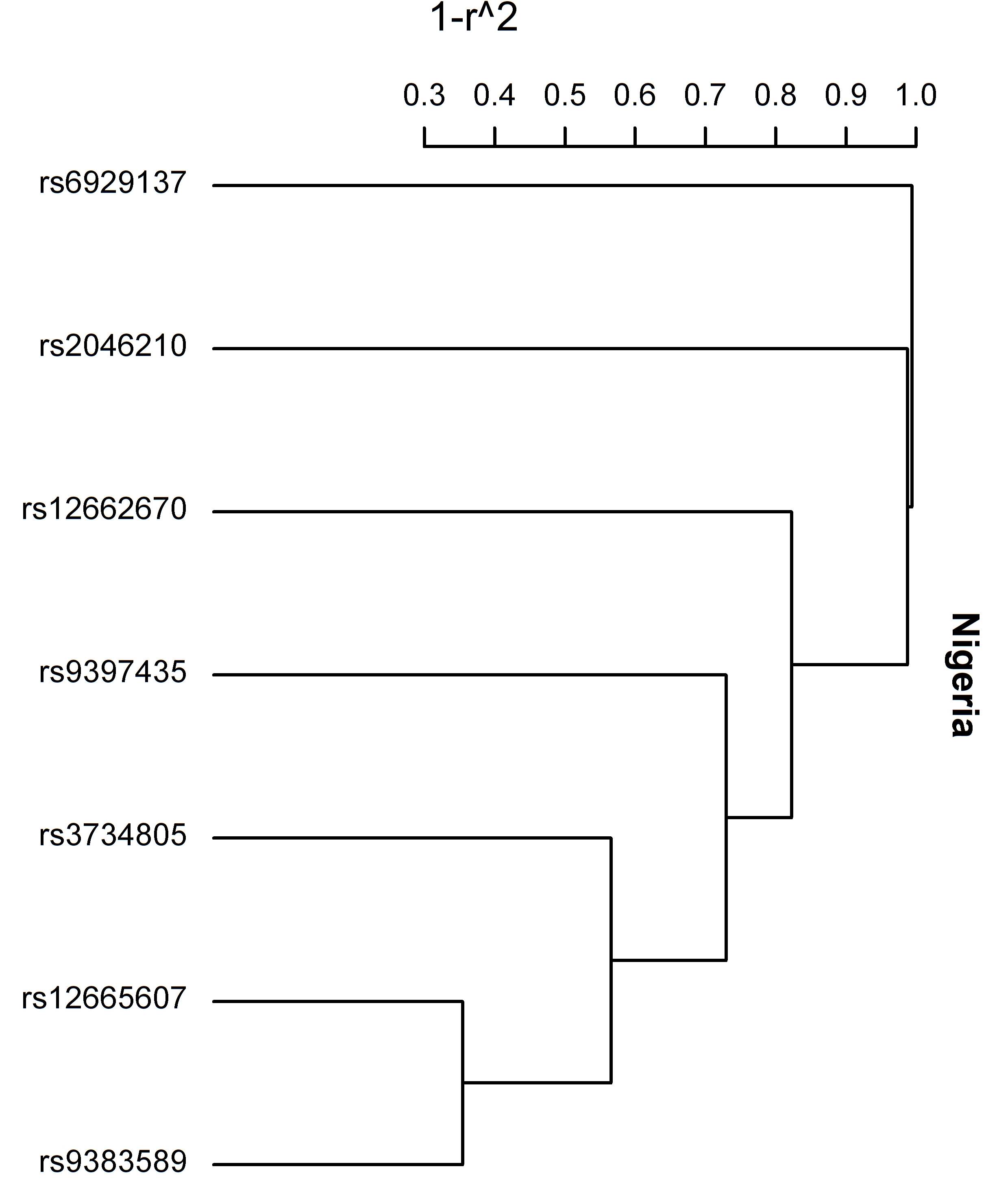

Supplement: Figure S3 — Dendrograms showing r2 relationships between the C6orf9-ESR1 SNPs genotyped in each study population. On the left are listed the SNPs that were genotyped in each of the study population samples. The name of the study population sample is indicated on the right of each panel. The SNPs are arranged in a hierarchical cluster dendrogram based on the r2 values between them derived from the observed genotypes for the SNPs. Note that the scales on the top of the panels show 1-r2 values (i.e. a value of 0 corresponds to an r2 of 1). The scale for the Taiwanese sample is limited in range between 0 and 0.4 (corresponding to an r2 range of 1 to 0.6) because all genotyped SNPs had r2 values greater than 0.6. The scale for the USA, African-American ancestry and the Nigerians ranges from 0.3 to 1.0 (corresponding to an r2 range of 0.7 to 0) because no pair of genotyped SNPs had r2 values between them of greater than 0.7. (2.35 MB DOC) [file pgen.1001029.s003.doc]

**Figure S4:**


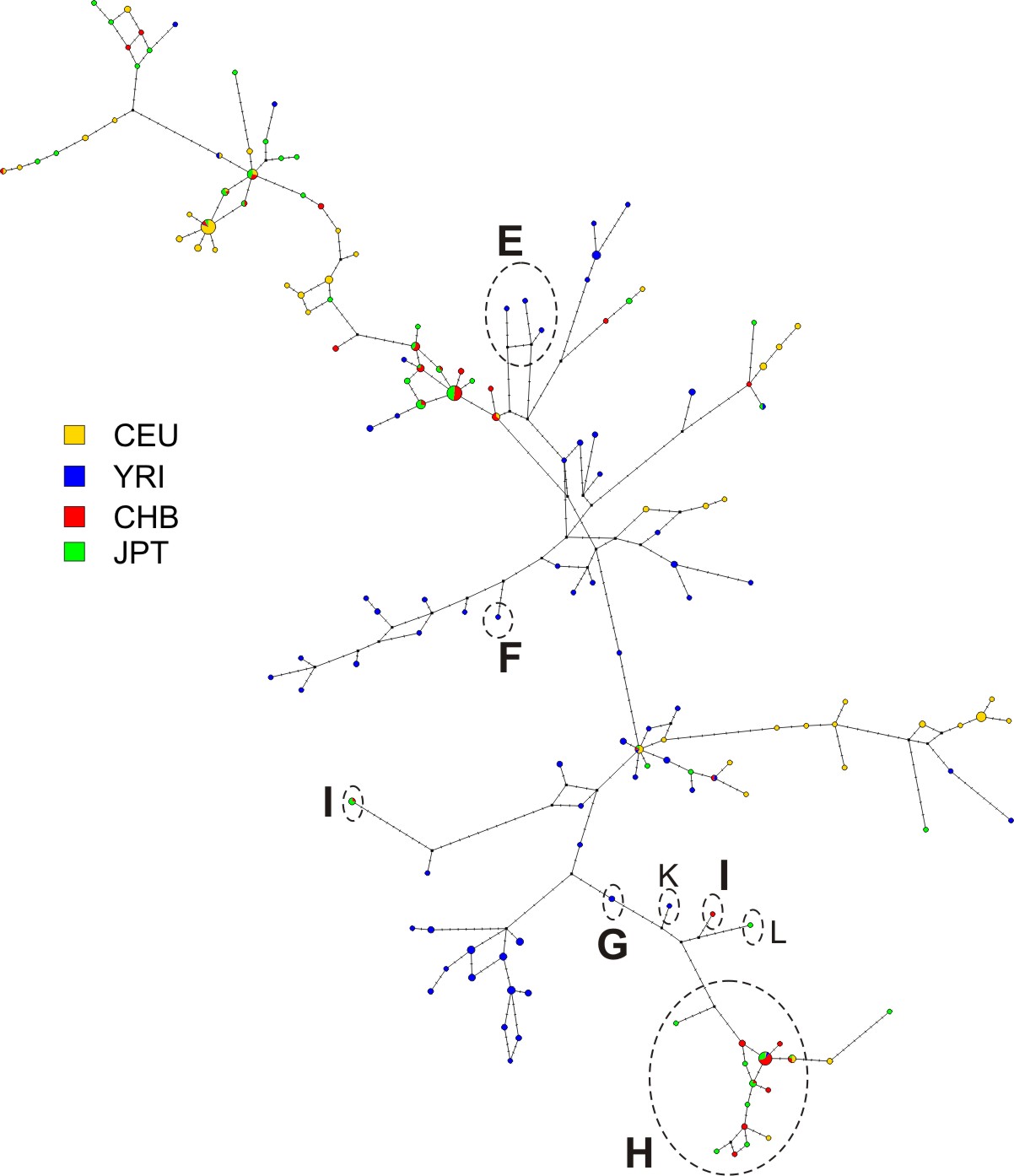

Supplement: Figure S4 — Haplotype analysis of the C6orf97-ESR1 region. Shown is a median joining (MJ) network describing the evolutionary relationships between haplotypes inferred from the genotypes of 81 HapMap (Phase II release 21) SNPs in the region chr6:151,950,821–151,992,990. Each haplotype is represented by a circle whose area reflects the overall number of copies observed and whose colour coding indicates the frequency of the haplotype in the different ancestral groups as indicated in the figure. Lines between the circles represent mutational evolutionary pathways between haplotypes reconstructed by the MJ algorithm. The line length is proportional to the number of inferred mutational differences between haplotypes. Black nodes represent non-sampled haplotypes that were reconstructed by the MJ algorithm as evolutionary intermediates between observed haplotypes. Encircled clusters of haplotypes are those carrying the rs9397435[G] allele and their lettering corresponds to the haplotype IDs shown in Table 3. Haplotypes K and L contain the rs9397435[G] allele but were too rare to qualify for inclusion in Table 3. Note that in Asians and Europeans, haplotypes bearing rs9397435[G] are clustered on the H, I and L branches, whereas in Yorubas the rs9397435[G] allele is found on more widely dispersed branches. Note also that Haplotype G, which was observed only in Yorubas, is in an ancestral position on the main H,I,L branch. (0.14 MB DOC) [file pgen.1001029.s004.doc]

**Figure S5:**

**
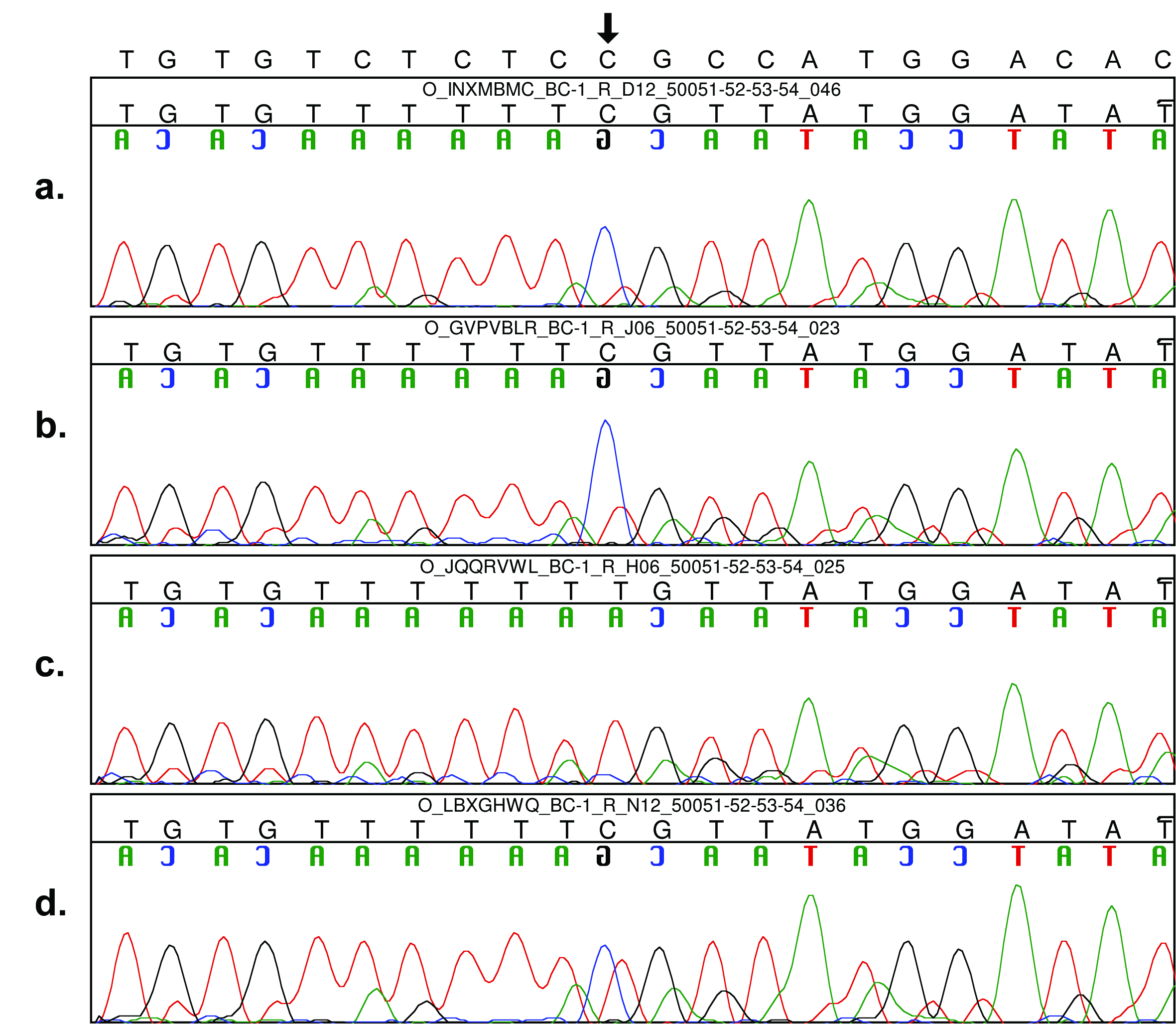
**

Supplement: Figure S5 — Bisulfite sequencing of region surrounding the C/T SNP at position 152,010,891 (arrowed) showing differential methylation of the C nucleotide in CC homozygotes. The top line shows the reference (non-bisulfite treated) sequence. Panels a–d show sequence traces of bisulfite-treated DNA from four CC homozygous individuals. In samples a and b the C nucleotide is predominantly methylated while a minority is unmethylated. In sample c, the C is predominantly unmethylated and in sample d similar amounts of methylated and unmethylated C are present. At neighboring C nucleotides, the conversion of unmethylated cytosine is complete, indicating that the bisulfite treatment was effective. In addition, we noted that nearby CpGs at positions 152,010,768, 152,010,842, 152,010,940, 152,011,003 and 152,011,013 were also methylated or partially methylated. (0.43 MB DOC) [file pgen.1001029.s005.doc]

**Figure S6:**

**
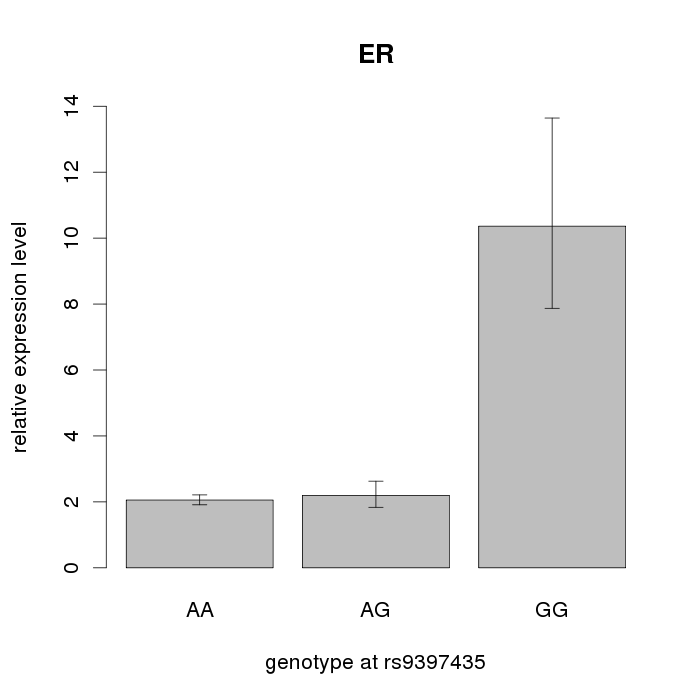

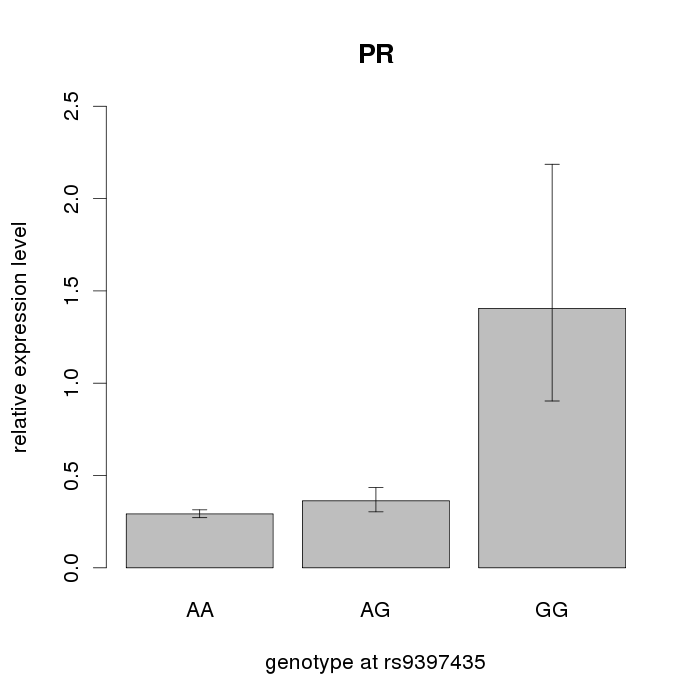

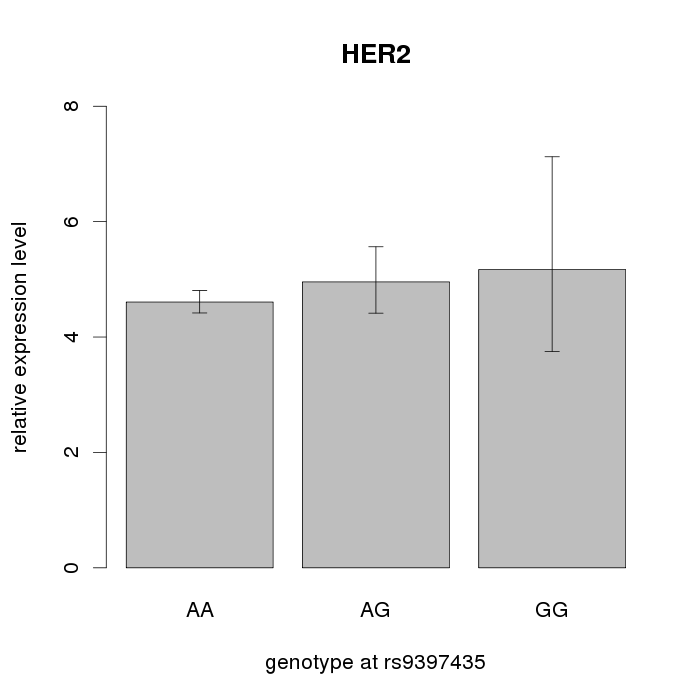
**

Supplement: Figure S6 — Quantitative RT-PCR analysis of ESR1 (ER), PGR (PG) and ERBB2 (HER2) mRNAs in tumours with different genotypes for rs9397435. RNA and DNA was isolated from 1,234 frozen tumour specimens. RNA levels were analyzed by RT-PCR and normalized to the mean level of three housekeeping genes. Relative expression levels are calculated as 2(mean Ct housekeeping−mean Ct target). Genotypes of rs9397435 were determined by Centaurus assay. Numbers of individuals with each genotype are 1,072 (AA), 151 (AG) and 11 (GG). Histogram displays the mean relative expression level (calculated as 10mean of log10 of relative expression level) for each genotype. Error bars indicate the standard error of the mean relative expression levels. (0.05 MB DOC) [file pgen.1001029.s006.doc]
