# Supplementary material for: Ancestry-Shift Refinement Mapping of the C6orf97-ESR1 Breast Cancer Susceptibility Locus
Source: PLoS Genet. 2010 Jul 22;6(7):e1001029. doi: 10.1371/journal.pgen.1001029 (PMC2908678; doi:10.1371/journal.pgen.1001029)
Supplement: Table S1 — Overview of the sample sets used in the study. (0.05 MB DOC) [file pgen.1001029.s007.doc]

| **Table S1: Overview of the sample sets used in the study** | | | | |  |
| --- | --- | --- | --- | --- | --- |
| **Sample Set** | **Ancestry** | **Cases** | **Controls** | **Type** | **Reference** |
| Iceland | European | 2638 | 3506 | Registry Ascertained Case: Population Based Control | a |
| U.S.A. (Mayo Clinic Breast Cancer Study [MCBCS]) | European | 1753 | 1487 | Clinic Ascertained Case: Population Based Control | b |
| Spain | European | 1009 | 1719 | Clinic Ascertained Case: Population Based Control | c |
| Netherlands (Nijmegen) | European | 727 | 1830 | Registry Ascertained Case: Population Based Control | c |
| Sweden (Stockholm) | European | 818 | 1750 | Clinic Ascertained Case: Population Based Control | d |
| Sweden (Northern) | European | 954 | 942 | Registry Ascertained Case: Population Based Control | e |
| Netherlands (Rotterdam) | European | 1792 | NA | Case only follow-up cohorts | f |
| Nigeria | African | 851 | 781 | Clinic Ascertained Case: Population Based Control | g |
| U.S.A. (Chicago) | African American | 300 | 153 | Clinic Ascertained Case: Hospital Based Control | h |
| Taiwan | Asian | 1126 | 1118 | Clinic Ascertained Case: Hospital Based Control | i |
| References: |  |  |  |  |  |
| ^a^ Stacey, S.N et al., PLoS Med. 2006 Jul;3(7):e217. | |  |  |  |  |
| ^b^ Olson, J.E. et al., Breast Cancer Res Treat. 2007 Apr;102(2):237-47. | | |  |  |  |
| ^c^ Stacey, S.N. et al., Nat Genet. 2007 Jul;39(7):865-9. | |  |  |  |  |
| ^d^ Margolin, S. et al., Genet Test. 2004 8(2):127-32. | |  |  |  |  |
| ^e^ Kaaks, R. et al., Cancer Causes Control. 2002 May;13(4):307-16. | | |  |  |  |
| ^f^ Hsieh, S.M. et al., Breast Cancer Res. 2009 Oct,11(5):R75 | | |  |  |  |
| ^g^ Huo, D. et al., Br J Cancer. 2008 Mar, 98(5):992-6. | | |  |  |  |
| ^h^ This paper |  |  |  |  |  |
| ^i^ Cheng, T.C. et al., Int J Cancer. 2005 Jan 20;113(3):345-53. | | |  |  |  |
