# Supplementary material for: Ancestry-Shift Refinement Mapping of the C6orf97-ESR1 Breast Cancer Susceptibility Locus
Source: PLoS Genet. 2010 Jul 22;6(7):e1001029. doi: 10.1371/journal.pgen.1001029 (PMC2908678; doi:10.1371/journal.pgen.1001029)
Supplement: Table S2 — Association of C6orf97/ESR1 SNPs with breast cancer in each population sample. (0.20 MB DOC) [file pgen.1001029.s008.doc]

| **Table S2: Association of C6orf97/ESR1 SNPs with Breast Cancer in each population sample** | | | | | | | | |
| --- | --- | --- | --- | --- | --- | --- | --- | --- |
| **Sample Set** | **Ancestry** | **SNP_allele** | **OR** | **P value** | **N Case** | **Freq Case** | **N Ctr** | **Freq Ctr** |
| Iceland | European | rs12662670_3 | 1.25 | 2.50E-03 | 2638 | 0.076 | 3506 | 0.062 |
| Iceland | European | rs12665607_1 | 1.28 | 7.90E-04 | 2638 | 0.076 | 3506 | 0.060 |
| Iceland | European | rs2046210_4 | 1.01 | 7.60E-01 | 2638 | 0.320 | 3506 | 0.317 |
| Iceland | European | rs3734804_1 | 0.98 | 5.90E-01 | 2638 | 0.468 | 3506 | 0.473 |
| Iceland | European | rs3734805_2 | 1.30 | 4.40E-04 | 2638 | 0.077 | 3506 | 0.060 |
| Iceland | European | rs6929137_1 | 1.03 | 4.80E-01 | 2638 | 0.302 | 3506 | 0.296 |
| Iceland | European | rs6932260_2 | 0.98 | 5.40E-01 | 2638 | 0.467 | 3506 | 0.473 |
| Iceland | European | rs7752591_1 | 0.98 | 5.20E-01 | 2638 | 0.473 | 3506 | 0.479 |
| Iceland | European | rs852003_1 | 1.04 | 3.50E-01 | 2638 | 0.564 | 3506 | 0.555 |
| Iceland | European | rs9383589_3 | 1.30 | 4.50E-04 | 2638 | 0.076 | 3506 | 0.060 |
| Iceland | European | rs9383932_3 | 1.13 | 3.20E-02 | 2638 | 0.132 | 3506 | 0.119 |
| Iceland | European | rs9397435_3 | 1.29 | 7.90E-04 | 2638 | 0.074 | 3506 | 0.059 |
| U.S.A. (MCBCS) | European | rs12662670_3 | 1.04 | 6.35E-01 | 1753 | 0.082 | 1487 | 0.079 |
| U.S.A. (MCBCS) | European | rs12665607_1 | 1.11 | 2.35E-01 | 1753 | 0.087 | 1487 | 0.079 |
| U.S.A. (MCBCS) | European | rs2046210_4 | 1.07 | 2.28E-01 | 1753 | 0.354 | 1487 | 0.340 |
| U.S.A. (MCBCS) | European | rs3734804_1 | 1.08 | 1.13E-01 | 1753 | 0.518 | 1487 | 0.499 |
| U.S.A. (MCBCS) | European | rs3734805_2 | 1.11 | 2.58E-01 | 1753 | 0.085 | 1487 | 0.077 |
| U.S.A. (MCBCS) | European | rs6929137_1 | 1.05 | 3.95E-01 | 1753 | 0.332 | 1487 | 0.322 |
| U.S.A. (MCBCS) | European | rs6932260_2 | 1.09 | 9.56E-02 | 1753 | 0.520 | 1487 | 0.499 |
| U.S.A. (MCBCS) | European | rs7752591_1 | 1.09 | 8.91E-02 | 1753 | 0.517 | 1487 | 0.495 |
| U.S.A. (MCBCS) | European | rs852003_1 | 1.07 | 1.81E-01 | 1753 | 0.588 | 1487 | 0.571 |
| U.S.A. (MCBCS) | European | rs9383589_3 | 1.09 | 3.44E-01 | 1753 | 0.084 | 1487 | 0.078 |
| U.S.A. (MCBCS) | European | rs9383932_3 | 1.05 | 5.20E-01 | 1753 | 0.145 | 1487 | 0.139 |
| U.S.A. (MCBCS) | European | rs9397435_3 | 1.16 | 1.15E-01 | 1753 | 0.078 | 1487 | 0.068 |
| Spain | European | rs12662670_3 | 1.16 | 1.14E-01 | 1009 | 0.099 | 1719 | 0.086 |
| Spain | European | rs12665607_1 | 1.14 | 1.61E-01 | 1009 | 0.106 | 1719 | 0.094 |
| Spain | European | rs2046210_4 | 1.11 | 7.24E-02 | 1009 | 0.400 | 1719 | 0.375 |
| Spain | European | rs3734805_2 | 1.11 | 2.58E-01 | 1009 | 0.102 | 1719 | 0.093 |
| Spain | European | rs6929137_1 | 1.11 | 8.23E-02 | 1009 | 0.377 | 1719 | 0.353 |
| Spain | European | rs7752591_1 | 1.09 | 1.27E-01 | 1009 | 0.595 | 1719 | 0.574 |
| Spain | European | rs852003_1 | 1.03 | 6.61E-01 | 1009 | 0.595 | 1719 | 0.589 |
| Spain | European | rs9383589_3 | 1.14 | 1.75E-01 | 1009 | 0.101 | 1719 | 0.090 |
| Spain | European | rs9383932_3 | 1.00 | 9.53E-01 | 1009 | 0.156 | 1719 | 0.157 |
| Spain | European | rs9397435_3 | 1.11 | 3.19E-01 | 1009 | 0.080 | 1719 | 0.073 |
| Netherlands (Nijmegen) | European | rs12662670_3 | 0.95 | 6.41E-01 | 727 | 0.080 | 1830 | 0.084 |
| Netherlands (Nijmegen) | European | rs12665607_1 | 0.93 | 5.10E-01 | 727 | 0.080 | 1830 | 0.085 |
| Netherlands (Nijmegen) | European | rs2046210_4 | 0.97 | 6.67E-01 | 727 | 0.345 | 1830 | 0.352 |
| Netherlands (Nijmegen) | European | rs3734805_2 | 0.94 | 5.56E-01 | 727 | 0.080 | 1830 | 0.086 |
| Netherlands (Nijmegen) | European | rs6929137_1 | 0.97 | 5.99E-01 | 727 | 0.317 | 1830 | 0.325 |
| Netherlands (Nijmegen) | European | rs7752591_1 | 1.07 | 2.67E-01 | 727 | 0.520 | 1830 | 0.503 |
| Netherlands (Nijmegen) | European | rs852003_1 | 1.06 | 3.91E-01 | 727 | 0.580 | 1830 | 0.567 |
| Netherlands (Nijmegen) | European | rs9383589_3 | 0.94 | 5.67E-01 | 727 | 0.081 | 1830 | 0.086 |
| Netherlands (Nijmegen) | European | rs9383932_3 | 1.08 | 3.48E-01 | 727 | 0.159 | 1830 | 0.148 |
| Netherlands (Nijmegen) | European | rs9397435_3 | 0.93 | 5.44E-01 | 727 | 0.073 | 1830 | 0.078 |
| Sweden (Stockholm) | European | rs12662670_3 | 1.11 | 3.93E-01 | 818 | 0.074 | 1750 | 0.067 |
| Sweden (Stockholm) | European | rs12665607_1 | 1.12 | 3.28E-01 | 818 | 0.076 | 1750 | 0.068 |
| Sweden (Stockholm) | European | rs2046210_4 | 1.07 | 2.71E-01 | 818 | 0.342 | 1750 | 0.327 |
| Sweden (Stockholm) | European | rs3734805_2 | 1.09 | 4.46E-01 | 818 | 0.074 | 1750 | 0.068 |
| Sweden (Stockholm) | European | rs6929137_1 | 1.07 | 3.14E-01 | 818 | 0.318 | 1750 | 0.304 |
| Sweden (Stockholm) | European | rs7752591_1 | 1.09 | 1.76E-01 | 818 | 0.499 | 1750 | 0.479 |
| Sweden (Stockholm) | European | rs852003_1 | 1.04 | 5.03E-01 | 818 | 0.565 | 1750 | 0.555 |
| Sweden (Stockholm) | European | rs9383589_3 | 1.20 | 1.35E-01 | 818 | 0.071 | 1750 | 0.060 |
| Sweden (Stockholm) | European | rs9383932_3 | 0.96 | 6.21E-01 | 818 | 0.125 | 1750 | 0.130 |
| Sweden (Stockholm) | European | rs9397435_3 | 1.16 | 2.09E-01 | 818 | 0.070 | 1750 | 0.060 |
| Sweden (Northern) | European | rs12662670_3 | 1.03 | 8.49E-01 | 954 | 0.046 | 942 | 0.044 |
| Sweden (Northern) | European | rs12665607_1 | 1.11 | 4.90E-01 | 954 | 0.052 | 942 | 0.047 |
| Sweden (Northern) | European | rs2046210_4 | 1.02 | 7.32E-01 | 954 | 0.314 | 942 | 0.309 |
| Sweden (Northern) | European | rs3734804_1 | 0.92 | 2.15E-01 | 954 | 0.431 | 942 | 0.451 |
| Sweden (Northern) | European | rs3734805_2 | 1.10 | 5.37E-01 | 954 | 0.051 | 942 | 0.047 |
| Sweden (Northern) | European | rs6929137_1 | 1.03 | 7.19E-01 | 954 | 0.303 | 942 | 0.297 |
| Sweden (Northern) | European | rs6932260_2 | 0.93 | 2.68E-01 | 954 | 0.433 | 942 | 0.451 |
| Sweden (Northern) | European | rs7752591_1 | 0.95 | 4.80E-01 | 954 | 0.428 | 942 | 0.440 |
| Sweden (Northern) | European | rs852003_1 | 0.96 | 5.50E-01 | 954 | 0.490 | 942 | 0.500 |
| Sweden (Northern) | European | rs9383589_3 | 1.13 | 4.37E-01 | 954 | 0.051 | 942 | 0.045 |
| Sweden (Northern) | European | rs9383932_3 | 1.09 | 4.47E-01 | 954 | 0.103 | 942 | 0.096 |
| Sweden (Northern) | European | rs9397435_3 | 1.00 | 9.92E-01 | 954 | 0.039 | 942 | 0.039 |
| Nigeria | African | rs12662670_3 | 1.36 | 1.65E-01 | 851 | 0.036 | 781 | 0.027 |
| Nigeria | African | rs12665607_1 | 1.76 | 7.90E-02 | 851 | 0.017 | 781 | 0.010 |
| Nigeria | African | rs2046210_4 | 1.00 | 9.60E-01 | 851 | 0.715 | 781 | 0.716 |
| Nigeria | African | rs3734805_2 | 1.33 | 1.59E-01 | 851 | 0.037 | 781 | 0.028 |
| Nigeria | African | rs6929137_1 | 0.97 | 6.74E-01 | 851 | 0.530 | 781 | 0.538 |
| Nigeria | African | rs9383589_3 | 1.66 | 4.54E-02 | 851 | 0.026 | 781 | 0.016 |
| Nigeria | African | rs9397435_3 | 1.39 | 1.60E-02 | 851 | 0.086 | 781 | 0.063 |
| U.S.A. (Chicago) | African American | rs12662670_3 | 2.12 | 3.49E-02 | 300 | 0.061 | 153 | 0.030 |
| U.S.A. (Chicago) | African American | rs12665607_1 | 2.47 | 7.54E-02 | 300 | 0.032 | 153 | 0.013 |
| U.S.A. (Chicago) | African American | rs2046210_4 | 0.92 | 5.72E-01 | 300 | 0.598 | 153 | 0.618 |
| U.S.A. (Chicago) | African American | rs3734805_2 | 1.13 | 7.04E-01 | 300 | 0.052 | 153 | 0.046 |
| U.S.A. (Chicago) | African American | rs6929137_1 | 1.19 | 2.12E-01 | 300 | 0.549 | 153 | 0.505 |
| U.S.A. (Chicago) | African American | rs9383589_3 | 1.47 | 3.71E-01 | 300 | 0.033 | 153 | 0.023 |
| U.S.A. (Chicago) | African American | rs9397435_3 | 1.20 | 4.83E-01 | 300 | 0.085 | 153 | 0.072 |
| Taiwan | Asian | rs12662670_3 | 1.22 | 2.46E-03 | 1126 | 0.343 | 1118 | 0.300 |
| Taiwan | Asian | rs12665607_1 | 1.24 | 6.15E-04 | 1126 | 0.372 | 1118 | 0.323 |
| Taiwan | Asian | rs2046210_4 | 1.24 | 4.26E-04 | 1126 | 0.415 | 1118 | 0.363 |
| Taiwan | Asian | rs3734804_1 | 1.21 | 1.44E-03 | 1126 | 0.470 | 1118 | 0.422 |
| Taiwan | Asian | rs3734805_2 | 1.22 | 2.01E-03 | 1126 | 0.368 | 1118 | 0.324 |
| Taiwan | Asian | rs6929137_1 | 1.15 | 2.53E-02 | 1126 | 0.380 | 1118 | 0.348 |
| Taiwan | Asian | rs6932260_2 | 1.23 | 7.71E-04 | 1126 | 0.473 | 1118 | 0.422 |
| Taiwan | Asian | rs7752591_1 | 1.25 | 2.99E-04 | 1126 | 0.475 | 1118 | 0.421 |
| Taiwan | Asian | rs852003_1 | 1.26 | 1.17E-04 | 1126 | 0.482 | 1118 | 0.424 |
| Taiwan | Asian | rs9383589_3 | 1.20 | 4.23E-03 | 1126 | 0.364 | 1118 | 0.323 |
| Taiwan | Asian | rs9383932_3 | 1.25 | 2.92E-04 | 1126 | 0.432 | 1118 | 0.378 |
| Taiwan | Asian | rs9397435_3 | 1.23 | 7.98E-04 | 1126 | 0.374 | 1118 | 0.326 |
