# Supplementary material for: Ancestry-Shift Refinement Mapping of the C6orf97-ESR1 Breast Cancer Susceptibility Locus
Source: PLoS Genet. 2010 Jul 22;6(7):e1001029. doi: 10.1371/journal.pgen.1001029 (PMC2908678; doi:10.1371/journal.pgen.1001029)
Supplement: Table S3 — Heterogeneity in risk estimates for combined population samples. (0.06 MB DOC) [file pgen.1001029.s009.doc]

| **Table S3: Heterogeneity in risk estimates for combined population samples** | | | |
| --- | --- | --- | --- |
| **Ancestry^a^** | **SNP_Allele** | ***P_het_*** | ***I^2^*** |
| All Ancestries | rs12662670_3 | 0.17 | 43.9 |
| All Ancestries | rs12665607_1 | 0.095 | 57.5 |
| All Ancestries | rs2046210_4 | 0.014 | 76.6 |
| All Ancestries | rs3734805_2 | 0.53 | 0 |
| All Ancestries | rs6929137_1 | 0.30 | 17.4 |
| All Ancestries | rs9383589_3 | 0.29 | 18.8 |
| All Ancestries | rs9397435_3 | 0.36 | 2 |
| European and Asian | rs3734804_1 | 3.4 x 10^-3^ | 88.4 |
| European and Asian | rs6932260_2 | 7.8 x 10^-4^ | 91.1 |
| European and Asian | rs7752591_1 | 3.1 x 10^-3^ | 88.6 |
| European and Asian | rs852003_1 | 3.0 x 10^-3^ | 88.7 |
| European and Asian | rs9383932_3 | 0.017 | 82.3 |
| All European | rs12662670_3 | 0.37 | 7.4 |
| All European | rs12665607_1 | 0.32 | 15.4 |
| All European | rs2046210_4 | 0.61 | 0 |
| All European | rs3734804_1 | 0.11 | 53.9 |
| All European | rs3734805_2 | 0.26 | 23.2 |
| All European | rs6929137_1 | 0.75 | 0 |
| All European | rs6932260_2 | 0.11 | 54.3 |
| All European | rs7752591_1 | 0.2 | 30.9 |
| All European | rs852003_1 | 0.87 | 0 |
| All European | rs9383589_3 | 0.26 | 22.9 |
| All European | rs9383932_3 | 0.65 | 0 |
| All European | rs9397435_3 | 0.29 | 19 |
| African and African American | rs12662670_3 | 0.29 | 10.5 |
| African and African American | rs12665607_1 | 0.57 | 0 |
| African and African American | rs2046210_4 | 0.63 | 0 |
| African and African American | rs3734805_2 | 0.68 | 0 |
| African and African American | rs6929137_1 | 0.19 | 40.5 |
| African and African American | rs9383589_3 | 0.81 | 0 |
| African and African American | rs9397435_3 | 0.62 | 0 |
| ^a^ Values for "All Ancestries" are the heterogeneity values that result from combining the risk estimate for the Taiwanese sample, the combined estimate for all European samples, and the combined estimate for African and African American samples. Values for "European and Asian" are the heterogeneity values that result from combining the risk estimate for the Taiwanese sample with the combined estimate for all European samples. Values for "All European" are the heterogeneity values that result from combining the risk estimates from all of the individual European sample sets. Values for "African and African American are the heterogeneity values that result from combining the risk estimates from the Nigerian and U.S.A. (Chicago) sample sets. | | | |
