# Supplementary material for: Ancestry-Shift Refinement Mapping of the C6orf97-ESR1 Breast Cancer Susceptibility Locus
Source: PLoS Genet. 2010 Jul 22;6(7):e1001029. doi: 10.1371/journal.pgen.1001029 (PMC2908678; doi:10.1371/journal.pgen.1001029)
Supplement: Table S4 — Non-HapMap SNPs in strong LD with rs9397435 identified from 1,000 Genomes Project Dataa. (0.05 MB DOC) [file pgen.1001029.s010.doc]

| **Table S4: Non-HapMap SNPs in strong LD with rs9397435 identified from 1000 Genomes Project Data^a^** | | | | | | | |
| --- | --- | --- | --- | --- | --- | --- | --- |
|  |  | **CEU** | | **YRI** | | **JPT/CHB** | |
| **dbSNP 130** | **Position B36** | **D´** | **r^2^** | **D´** | **r^2^** | **D´** | **r^2^** |
| not listed | 151989450 | 1.00 | 0.76 | nd^b^ | nd | 1.00 | 0.96 |
| rs9397437 | 151994025 | 1.00 | 0.85 | 1.00 | 0.53 | 1.00 | 1.00 |
| rs58343273 | 151994873 | 1.00 | 1.00 | 0.67 | 0.04 | 1.00 | 1.00 |
| rs9383590 | 151995458 | 1.00 | 1.00 | nd | nd | 1.00 | 1.00 |
| rs60954078 | 151997607 | 1.00 | 1.00 | nd | nd | nd | nd |
| rs12173562 | 151999263 | 1.00 | 1.00 | nd | nd | 1.00 | 1.00 |
| rs6912323 | 152000305 | 1.00 | 1.00 | 0.49 | 0.02 | 1.00 | 1.00 |
| not listed | 152010891 | 1.00 | 1.00 | nd | nd | 1.00 | 0.96 |
| rs9371545 | 152011433 | 1.00 | 1.00 | nd | nd | 1.00 | 0.96 |
| rs9479091 | 152031302 | 1.00 | 0.76 | nd | nd | nd | nd |
| ^a^ Data were obtained from the 1000 Genomes project April 2009 release (ftp://ftp.1000genomes.ebi.ac.uk/) which includes data on 57 individuals of European ancestry (CEU), 56 Yorubas (YRI) and 59 Japanese or Han Chinese (JPT/CHB). SNPs were included in the table if they had an r^2^ value of >0.75 with rs9397435 in both CEU and JPT/CHB or an r^2^ value of >0.75 in one of the two ancestries and were missing data for the other. ^b^ nd, no data available. | | | | | | | |
