# Supplementary material for: Ancestry-Shift Refinement Mapping of the C6orf97-ESR1 Breast Cancer Susceptibility Locus
Source: PLoS Genet. 2010 Jul 22;6(7):e1001029. doi: 10.1371/journal.pgen.1001029 (PMC2908678; doi:10.1371/journal.pgen.1001029)
Supplement: Table S5 — LD relations with the novel chr6:152,010,891[C/T] SNP (rs77275268). (0.04 MB DOC) [file pgen.1001029.s011.doc]

| **Table S5: LD relations with the novel chr6:152,010,891[C/T] SNP (rs77275268)** | | | | |
| --- | --- | --- | --- | --- |
| **Sample set** | **SNP1** | **SNP 2** | **D´** | **r^2^** |
| Iceland | rs77275268 | rs9397435 | 1.00 | 0.95 |
| Taiwan | rs77275268 | rs9397435 | 0.98 | 0.94 |
| Nigeria | rs77275268 | rs9397435 | 0.98 | 0.23 |
| Nigeria | rs77275268 | rs12662670 | 0.72 | 0.31 |
| Nigeria | rs77275268 | rs12665607 | 1.00 | 0.74 |
| Nigeria | rs77275268 | rs9383589 | 0.94 | 0.76 |
| Nigeria | rs77275268 | rs3734805 | 0.94 | 0.50 |
| Nigeria | rs77275268 | rs6929137 | 1.00 | 0.02 |
| Nigeria | rs77275268 | rs2046210 | 1.00 | 0.01 |
