# Supplementary material for: Ancestry-Shift Refinement Mapping of the C6orf97-ESR1 Breast Cancer Susceptibility Locus
Source: PLoS Genet. 2010 Jul 22;6(7):e1001029. doi: 10.1371/journal.pgen.1001029 (PMC2908678; doi:10.1371/journal.pgen.1001029)
Supplement: Table S6 — Levels of ERα, PR, and HER2 mRNA in primary tumours, stratified by rs9397435 genotype and assessed under multiplicative and recessive inheritance models. (0.05 MB DOC) [file pgen.1001029.s012.doc]

| **Table S6: Levels of ERα, PR and HER2 mRNA in primary tumours, stratified by rs9397435 genotype and assessed under multiplicative and recessive inheritance models** | | | | | | | | | | | | |
| --- | --- | --- | --- | --- | --- | --- | --- | --- | --- | --- | --- | --- |
| **mRNA** | **Value Type** | **Multiplicative Model** | | | **Full Genotype Model** | | | **Recessive Model** | | | **Full vs Multiplicative Model** | **Full vs Recessive Model** |
|  | Genotypes^a^ | **AA** | **AG** | **GG** | **AA** | **AG** | **GG** | **AA** | **AG** | **GG** |  |  |
| *ESR1* | Effect^b^ | 1.00 (ref) | 1.28 | 1.63 | 1.00 (ref) | 1.07 | 4.99 | 1.00 (ref) | 1.00 | 5.00 |  |  |
|  | *P^c^* |  | 0.177 |  |  | 0.075 |  |  | 0.024 |  | 0.067 | 0.750 |
| *PGR* | Effect^b^ | 1.00 (ref) | 1.42 | 2.02 | 1.00 (ref) | 1.24 | 4.80 | 1.00 (ref) | 1.00 | 4.69 |  |  |
|  | *P^c^* |  | 0.050 |  |  | 0.055 |  |  | 0.031 |  | 0.161 | 0.289 |
| *ERBB2* | Effect^b^ | 1.00 (ref) | 1.07 | 1.15 | 1.00 (ref) | 1.08 | 1.12 | 1.00 (ref) | 1.00 | 1.18 |  |  |
|  | *P^c^* |  | 0.512 |  |  | 0.805 |  |  | 0.801 |  | 0.962 | 0.543 |
| ^a^ Numbers of tumours with each genotype are 1072 (AA), 151 (AG) and 11 (GG). ^b^ The fold-effect on expression for each genotype, compared to the expression level in the AA genotype ^c^ Significance calculated under the models indicated after log_10_ transformation of the expression levels. | | | | | | | | | | | | |
