# Supplementary material for: Ancestry-Shift Refinement Mapping of the C6orf97-ESR1 Breast Cancer Susceptibility Locus
Source: PLoS Genet. 2010 Jul 22;6(7):e1001029. doi: 10.1371/journal.pgen.1001029 (PMC2908678; doi:10.1371/journal.pgen.1001029)
Supplement: Table S7 — Stratification by clinical variables of breast cancer associations with rs9397435[G] in combined European ancestry population samplesa. (0.11 MB DOC) [file pgen.1001029.s013.doc]

| **Table S7: Stratification by clinical variables of breast cancer associations with rs9397435[G] in combined European ancestry population samples^a^** | | | | | | | | | |
| --- | --- | --- | --- | --- | --- | --- | --- | --- | --- |
|  | **Number of Contributing Sample Sets** | **Class 1** | | **Class 2** | |  |  |  |  |
| **Comparison (Class 1 vs Class 2)** |  | **Number** | **Frequency** | **Number** | **Frequency** | **OR** | **(95% CI)** | ***P*** | ***P_het_*** |
| ER negative vs Control | 6 | 1128 | 0.075 | 11228 | 0.063 | 1.30 | (1.10, 1.53) | 2.5 x 10^-3^ | 0.75 |
| ER positive vs Control | 6 | 4310 | 0.071 | 11228 | 0.063 | 1.17 | (1.06, 1.29) | 2.4 x 10^-3^ | 0.10 |
| ER positive vs ER negative^b^ | 7 | 5611 | 0.071 | 1568 | 0.076 | 0.91 | (0.78, 1.05) | 0.20 | 0.95 |
|  |  |  |  |  |  |  |  |  |  |
| PR negative vs Control | 6 | 1631 | 0.075 | 11228 | 0.063 | 1.25 | (1.08, 1.44) | 2.5 x 10^-3^ | 0.85 |
| PR positive vs Control | 6 | 3728 | 0.071 | 11228 | 0.063 | 1.18 | (1.06, 1.31) | 2.8 x 10^-3^ | 0.24 |
| PR positive vs PR negative^b^ | 7 | 4806 | 0.072 | 2192 | 0.074 | 0.97 | (0.84, 1.11) | 0.62 | 0.35 |
|  |  |  |  |  |  |  |  |  |  |
| HER2 negative vs Control | 4 | 1733 | 0.075 | 8537 | 0.069 | 1.11 | (0.96, 1.29) | 0.15 | 0.66 |
| HER2 positive vs Control | 4 | 947 | 0.081 | 8537 | 0.069 | 1.19 | (0.99, 1.42) | 0.063 | 0.11 |
| HER2 positive vs Negative^b^ | 5 | 1138 | 0.079 | 2777 | 0.073 | 1.09 | (0.89, 1.32) | 0.40 | 0.84 |
|  |  |  |  |  |  |  |  |  |  |
| Triple Negative No vs Control | 4 | 4042 | 0.080 | 8537 | 0.069 | 1.19 | (1.07, 1.32) | 9.0 x 10^-4^ | 0.07 |
| Triple Negative Yes vs Control | 4 | 265 | 0.078 | 8537 | 0.069 | 1.26 | (0.91, 1.74) | 0.17 | 0.48 |
| Triple Negative Yes vs No^b^ | 5 | 437 | 0.080 | 4042 | 0.077 | 1.10 | (0.87, 1.41) | 0.42 | 0.89 |
|  |  |  |  |  |  |  |  |  |  |
| In Situ Tumour vs Control | 3 | 566 | 0.071 | 6818 | 0.068 | 1.08 | (0.85, 1.38) | 0.52 | 0.53 |
| Invasive Tumour vs Control | 6 | 5837 | 0.069 | 11228 | 0.063 | 1.16 | (1.06, 1.27) | 1.6 x 10^-3^ | 0.20 |
| Invasive vs In Situ Tumour | 3 | 4163 | 0.076 | 566 | 0.071 | 1.11 | (0.87, 1.41) | 0.41 | 0.30 |
|  |  |  |  |  |  |  |  |  |  |
| Stage 1 vs Control | 6 | 2659 | 0.063 | 11228 | 0.063 | 1.10 | (0.97, 1.25) | 0.14 | 0.14 |
| Stage 2 vs Control | 6 | 2109 | 0.072 | 11228 | 0.063 | 1.19 | (1.04, 1.35) | 0.011 | 0.96 |
| Stage 3&4 vs Control | 6 | 748 | 0.068 | 11228 | 0.063 | 1.25 | (1.02, 1.53) | 0.029 | 0.16 |
|  |  |  |  |  |  |  |  |  |  |
| Node Negative vs Control^c^ | 6 | 3523 | 0.063 | 11228 | 0.063 | 1.11 | (0.99, 1.24) | 0.08 | 0.04 |
| Node Positive vs Control^c^ | 6 | 2053 | 0.077 | 11228 | 0.063 | 1.25 | (1.10, 1.42) | 9.1 x 10^-4^ | 0.51 |
| Node Positive vs Node Negative^b,c^ | 7 | 2718 | 0.077 | 4592 | 0.066 | 1.10 | (0.94, 1.28) | 0.23 | 0.05 |
|  |  |  |  |  |  |  |  |  |  |
| Differentiation Grade 1 vs Control | 6 | 1021 | 0.066 | 11228 | 0.063 | 1.07 | (0.88, 1.29) | 0.5 | 0.25 |
| Differentiation Grade 2 vs Control | 6 | 2039 | 0.073 | 11228 | 0.063 | 1.17 | (1.02, 1.34) | 0.027 | 0.24 |
| Differentiation Grade 3 vs Control | 6 | 1386 | 0.075 | 11228 | 0.064 | 1.25 | (1.07, 1.47) | 4.6 x 10^-3^ | 0.2 |
|  |  |  |  |  |  |  |  |  |  |
| Invasive Ductal Carcinoma vs Control | 5 | 3834 | 0.080 | 10287 | 0.067 | 1.22 | (1.10, 1.36) | 1.8 x 10^-4^ | 0.078 |
| Invasive Lobular Carcinoma vs Control | 5 | 588 | 0.068 | 10287 | 0.068 | 1.11 | (0.88, 1.40) | 0.38 | 0.79 |
| Other Invasive Histology vs Control | 5 | 598 | 0.075 | 10287 | 0.067 | 1.15 | (0.91, 1.45) | 0.24 | 0.26 |
| Invasive Ductal vs Lobular^b^ | 6 | 4960 | 0.770 | 740 | 0.069 | 1.11 | (0.92, 1.34) | 0.26 | 0.41 |
|  |  |  |  |  |  |  |  |  |  |
| **Trend tests:** | **Number of Contributing Sample Sets** | **Number** | **Beta** | **(95% CI)** | ***P*** | ***P_het_*** |  |  |  |
| Stage 1 to 4^b^ | 7 | 7190 | 0.03 | (-0.02, 0.07) | 0.22 | 0.67 |  |  |  |
| Grade 1 to 3^b^ | 7 | 5635 | 0.03 | (-0.02, 0.08) | 0.2 | 0.26 |  |  |  |
| Age at first invasive breast cancer^b^ | 7 | 7450 | -0.97 | (-1.74, -0.19) | 0.015 | 0.8 |  |  |  |
| ^a^ All analyses are univariate. ^b^ Includes data from the Netherlands (Rotterdam) case-only sample set. ^c^ For tumours stage 1-4 | | | | | | | | | |
