# Supplementary material for: Ancestry-Shift Refinement Mapping of the C6orf97-ESR1 Breast Cancer Susceptibility Locus
Source: PLoS Genet. 2010 Jul 22;6(7):e1001029. doi: 10.1371/journal.pgen.1001029 (PMC2908678; doi:10.1371/journal.pgen.1001029)
Supplement: Table S8 — Stratification by clinical variables of breast cancer associations with rs9397435[G] in Taiwana (0.11 MB DOC) [file pgen.1001029.s014.doc]

| **Table S8: Stratification by clinical variables of breast cancer associations with rs9397435[G] in Taiwan^a^** | | | | | | | |
| --- | --- | --- | --- | --- | --- | --- | --- |
|  | **Class 1** | | **Class 2** | |  |  |  |
| **Comparison (Class 1 vs Class 2)** | **Number** | **Frequency** | **Number** | **Frequency** | **OR** | **(95% CI)** | ***P*** |
| ER negative vs Control | 349 | 0.368 | 1118 | 0.326 | 1.20 | (1.00, 1.43) | 0.045 |
| ER positive vs Control | 667 | 0.375 | 1118 | 0.326 | 1.24 | (1.08, 1.43) | 2.6 x 10^-3^ |
| ER positive vs ER negative | 667 | 0.3765 | 349 | 0.368 | 1.04 | (0.85, 1.27) | 0.70 |
|  |  |  |  |  |  |  |  |
| PR negative vs Control | 364 | 0.375 | 1118 | 0.326 | 1.24 | (1.04, 1.48) | 0.017 |
| PR positive vs Control | 649 | 0.371 | 1118 | 0.326 | 1.22 | (1.06, 1.41) | 0.007 |
| PR positive vs PR negative | 649 | 0.371 | 364 | 0.375 | 0.99 | (0.87, 1.13) | 0.88 |
|  |  |  |  |  |  |  |  |
| HER2 negative vs Control | 376 | 0.377 | 1118 | 0.326 | 1.25 | (1.05, 1.48) | 0.01 |
| HER2 positive vs Control | 150 | 0.349 | 1118 | 0.326 | 1.10 | (0.86, 1.41) | 0.45 |
| HER2 positive vs Negative | 150 | 0.349 | 376 | 0.379 | 0.88 | (0.66, 1.17) | 0.38 |
|  |  |  |  |  |  |  |  |
| Triple Negative No vs Control | 810 | 0.37 | 1118 | 0.326 | 1.21 | (1.06, 1.38) | 5.4 x 10^-3^ |
| Triple Negative Yes vs Control | 100 | 0.352 | 1118 | 0.326 | 1.11 | (0.83, 1.48) | 0.48 |
| Triple Negative Yes vs No | 100 | 0.352 | 810 | 0.37 | 0.93 | (0.69, 1.26) | 0.64 |
|  |  |  |  |  |  |  |  |
| In Situ Tumour vs Control | 102 | 0.406 | 1118 | 0.326 | 1.40 | (1.04, 1.88) | 0.026 |
| Invasive Tumour vs Control | 923 | 0.372 | 1118 | 0.326 | 1.22 | (1.07, 1.39) | 2.3 x 10^-3^ |
| Invasive vs In Situ Tumour | 923 | 0.372 | 102 | 0.406 | 0.87 | (0.65, 1.17) | 0.36 |
|  |  |  |  |  |  |  |  |
| Stage 1 vs Control | 400 | 0.395 | 1118 | 0.326 | 1.35 | (1.14, 1.60) | 4.8 x 10^-4^ |
| Stage 2 vs Control | 368 | 0.348 | 1118 | 0.326 | 1.10 | (0.92, 1.31) | 0.289 |
| Stage 3&4 vs Control | 155 | 0.372 | 1118 | 0.326 | 1.22 | (0.95, 1.57) | 0.117 |
|  |  |  |  |  |  |  |  |
| Node Negative vs Control^b^ | 525 | 0.362 | 1118 | 0.326 | 1.18 | (1.01, 1.38) | 0.038 |
| Node Positive vs Control^b^ | 333 | 0.356 | 1118 | 0.326 | 1.14 | (0.95, 1.36) | 0.15 |
| Node Positive vs Node Negative^b^ | 333 | 0.359 | 525 | 0.363 | 0.98 | (0.79, 1.21) | 0.85 |
|  |  |  |  |  |  |  |  |
| Differentiation Grade 1 vs Control | 122 | 0.356 | 1118 | 0.326 | 1.14 | (0.86, 1.51) | 0.357 |
| Differentiation Grade 2 vs Control | 449 | 0.38 | 1118 | 0.326 | 1.27 | (1.08, 1.49) | 3.8 x 10^-3^ |
| Differentiation Grade 3 vs Control | 278 | 0.36 | 1118 | 0.326 | 1.16 | (0.95, 1.42) | 0.14 |
|  |  |  |  |  |  |  |  |
| Invasive Ductal Carcinoma vs Control | 783 | 0.371 | 1118 | 0.326 | 1.21 | (1.06, 1.39) | 5.8 x 10^-3^ |
| Invasive Lobular Carcinoma vs Control | 46 | 0.315 | 1118 | 0.326 | 0.95 | (0.63, 1.44) | 0.81 |
| Other Invasive Histology vs Control | 87 | 0.383 | 1118 | 0.327 | 1.28 | (0.93, 1.77) | 0.14 |
|  |  |  |  |  |  |  |  |
| **Trend tests:** | **Number** | **Beta** | **(95% CI)** | ***P*** |  |  |  |
| Stage 1 to 4 | 923 | -0.0454 | (-0.12, 0.03) | 0.208 |  |  |  |
| Grade 1 to 3 | 849 | -0.0141 | (-0.08, 0.05) | 0.685 |  |  |  |
| Age at first invasive breast cancer | 923 | 0.39 | (-0.64, 1.43) | 0.45 |  |  |  |
| ^a^ All analyses are univariate. ^b^ For tumours stage 1-4. | | | | | | | |
